# Cues to Stress Assignment in Reading Aloud

**DOI:** 10.1037/xge0000380

**Published:** 2018-01

**Authors:** Maria Ktori, Petroula Mousikou, Kathleen Rastle

**Affiliations:** 1 Department of Psychology, Royal Holloway, University of London

**Keywords:** reading aloud, stress assignment, sublexical cues, computational modeling

## Abstract

Research seeking to uncover the mechanisms by which we read aloud has focused almost exclusively on monosyllabic items presented in isolation. Consequently, important challenges that arise when considering polysyllabic word reading, such as stress assignment, have been ignored, while little is known about how important sentence-level stress cues, such as syntax and rhythm, may influence word reading aloud processes. The present study seeks to fill these gaps in the literature by (a) documenting the individual influences of major sublexical cues that readers use to assign stress in single-word reading in English and (b) determining how these cues may interact with contextual stress factors in sentence reading. In Experiments 1, 2, and 3 we investigated the effects of prefixation, orthographic weight (i.e., number of letters in a syllable), and vowel length on stress assignment by asking participants to read aloud carefully-constructed nonwords that varied on the presence of these cues. Results revealed individual effects of all three cues on the assignment of second-syllable stress. In Experiment 4, we tested the effects of these cues on stress assignment in the context of sentence reading. Results showed that sublexical cues influenced stress assignment over and above higher-level syntactic and rhythmic cues. We consider these findings in the framework of extant rule-based, distributed-connectionist, and Bayesian approaches to stress assignment in reading aloud, and we discuss their applications to understanding reading development and acquired and developmental reading disorders.

One of the fundamental insights of psycholinguistic research over the past 40 years is that the computation of sound-based (phonological) codes is central to skilled reading and reading acquisition (see e.g., Frost, 1998; Melby-Lervåg, Lyster, & Hulme, 2012; Perfetti, 2003; Rastle & Brysbaert, 2006; Share, 1995 for reviews). This insight has motivated an extensive body of empirical research (e.g., Ferrand & Grainger, 1992; Lukatela & Turvey, 1994) and the development of computational models (e.g., Coltheart, Rastle, Perry, Langdon, & Ziegler, 2001; Harm & Seidenberg, 2004; Perry, Ziegler, & Zorzi, 2007), which seek to explain how we translate printed letter strings into their corresponding sounds. It has also supported major shifts in approaches to reading instruction, so that children’s learning of the relationship between letters and sounds (i.e., phonics) is given high priority (e.g., Rayner, Foorman, Perfetti, Pesetsky, & Seidenberg, 2001; Rose, 2006).

Despite widespread acceptance of the central role of phonology in reading, research seeking to uncover the mechanisms by which we translate orthography to phonology has focused almost exclusively on monosyllables. Critically, the focus on monosyllables has allowed reading research to ignore major challenges that emerge when considering polysyllables, the most important of which is the assignment of stress. Stress at the level of the single word refers to the phonetic accentuation of a particular syllable (as in *ca*mel vs. ca*nal*). Evidence from eye-movement research suggests that the computation of stress facilitates access to lexical information in silent reading (Ashby & Clifton, 2005). Stress is also used to disambiguate words phonologically in sentence contexts (e.g., *record* as a noun or verb), and at the word level (e.g., trus*tee* vs. *tru*sty). More importantly, polysyllables make up the vast majority of words in most languages (e.g., Baayen, Piepenbrock, & Gulikers, 1995 for English, Dutch, and German languages); thus, failing to understand how phonology is computed for polysyllables presents a major impediment to any theory of reading that aspires to completeness.

The present study provides a substantial advance in our understanding of how we read letter strings with more than one syllable. In a series of three reading aloud experiments, we investigate the major factors that influence stress assignment at the single-word level. The data from these experiments are then used to assess the performance of three computational accounts of reading disyllables aloud—the rule-based disyllabic algorithm of Rastle and Coltheart (2000), the connectionist dual process (CDP++) model (Perry, Ziegler, & Zorzi, 2010), and the connectionist print-to-stress network of Ševa, Monaghan, and Arciuli (2009). We also consider these results in the context of a Bayesian approach to understanding stress assignment (Jouravlev & Lupker, 2015a). In a fourth experiment, we test the influence of higher-level contextual cues on stress assignment, and critically, assess how these sentence-level cues impact on the word-level cues revealed in the first three experiments. This fourth experiment is especially interesting because it provides a means to begin to bridge the empirical evidence from single-word reading aloud into the domain of sentence reading. Research on single-word reading aloud and sentence reading span vast literatures; however, we show that these literatures rarely overlap. This experiment will reveal whether the mechanisms underlying stress assignment in single-word reading aloud are fundamentally altered when printed stimuli are placed in sentence contexts. Our data will thus provide vital new constraints on the development of computational models of reading aloud as they move beyond the monosyllabic domain, and beyond the domain of words read in isolation.

## Stress Assignment in Single-Word Reading

Rastle and Coltheart (2000) considered how an existing model of reading aloud, namely the *dual-route cascaded model* (hereafter referred to as DRC; Coltheart et al., 2001), could accommodate reading of disyllables in English, including the assignment of stress. One possibility is that stress could be retrieved lexically, using whole-word information stored in memory. This mechanism would allow the reader to pronounce familiar words such as *camel* and *canal*, which look similar but are stressed on different syllables. However, this mechanism would not account for readers’ ability to assign stress to unfamiliar words or nonwords that are not stored in lexical memory (Rastle & Coltheart, 2000). Thus, another possibility is that stress could be computed on the basis of sublexical information, much as the dual-route theory asserts that people are able to compute the phonemes of a printed nonword. So how might stress be computed without recourse to lexical information?

### Distribution of Stress Patterns in the Language

In considering what might be the nature of the sublexical cues used to assign stress, one of the first proposals put forward was that readers assign stress on the basis of the simple distribution of stress patterns in the language (Colombo, 1992). For example, about 80% of Italian polysyllabic words are stressed on the penultimate syllable (Sulpizio, Burani, & Colombo, 2015), so a sublexical process applicable to polysyllabic words could implement penultimate stress as a default. Similarly, around 75% of English disyllables are stressed on the first syllable (calculated from CELEX, Baayen et al., 1995), so a sublexical process that implemented initial stress as a default would stress a high proportion of words correctly. Based on this type of language-specific distributional rule, words that follow the rule would be considered regular, whereas words that fail to adhere to the default stress rule would be considered irregular.

However, the evidence to support this hypothesis is weak. Although some studies in Italian and Russian report a processing advantage for stress-regular words based on the distributional rule (Colombo, 1992; Colombo & Tabossi, 1992 in Italian; Jouravlev & Lupker, 2014, in Russian in the by-subjects analysis) others fail to do so (Burani & Arduino, 2004; Burani, Paizi, & Sulpizio, 2014, in Italian; Jouravlev & Lupker, 2014, in Russian in the by-items analysis). In English, the strongest evidence for a distributional rule comes from a study carried out by Brown, Lupker, and Colombo (1994). Using items developed by Monsell, Doyle, and Haggard (1989), they reported that disyllabic words stressed on the first syllable were read aloud faster than disyllabic words stressed on the second syllable. However, analyses were conducted only by subjects, so it is unknown whether this effect held across items. Similarly, although the earlier study by Monsell et al. (1989) had reported a numerical advantage for disyllables stressed on the first syllable, no statistics relevant to this comparison were reported. In a more recent study of English reading aloud, Rastle and Coltheart (2000) failed to find any evidence for a stress regularity effect based on the distributional rule. Stress regularity effects were absent even for low-frequency words, in which sublexical information is thought to play a more potent role in the translation of orthography to phonology (Jared & Seidenberg, 1990; Seidenberg, Waters, Barnes, & Tanenhaus, 1984). Hence, the empirical support for an account of stress assignment based on the distribution of stress patterns in the language is weak.

### Distribution of “Word Endings” and “Word Beginnings”

Some authors have suggested instead that distributional information about the relationship between smaller sublexical units and stress patterns may influence stress assignment. There is reasonably good evidence that the “endings” of words can serve as an indicator of stress position in Italian. The *ending* in this research is defined as the orthographic unit that includes all the final letters of a word starting from the nucleus of the second syllable (e.g., *-ola* in *picola*). Words that contain the same endings are said to be part of the same “stress neighborhood.” Several studies have now indicated that the reading aloud of Italian words and nonwords is influenced by the consistency of items within their stress neighborhood (see Sulpizio et al., 2015, for a review). Words and nonwords with many stress friends (i.e., items with a stress pattern that is consistent with the stress pattern of the majority of words in the same neighborhood) are read faster and more accurately than those with many stress enemies (i.e., items with a stress pattern that is inconsistent with that of most words in the same neighborhood). Furthermore, this effect appears to be modulated by the number of stress neighbors that are available in the language for a given ending, with a greater influence being observed for stress neighbors that are widely represented in the Italian language (Sulpizio, Arduino, Paizi, & Burani, 2013). Finally, Jouravlev and Lupker (2014, 2015b) provided evidence that the orthographic endings of words can serve as a stress cue in Russian.

The evidence that distributional properties of word endings can influence stress assignment is less plentiful in English. The most rigorous study investigating this hypothesis was conducted by Arciuli and Cupples (2006). They reported a linguistic analysis of 340 endings in disyllabic English words and showed that these are associated to varying degrees with particular stress patterns (e.g., the endings *-ock* and *-ibe* are associated with first- and second-syllable stress, respectively). They then demonstrated that the presence of these endings in nonwords biased stress decisions when adults were asked to underline the part of a nonword that they would emphasize had they been reading the nonword aloud. Subsequently, Arciuli and Cupples (2007) provided preliminary data on how distributional properties of word beginnings (i.e., letter string up to and including the first vowel or vowel cluster) might also impact adults’ stress decisions.

Despite recent enthusiasm for the notion that simple units like word beginnings and word endings may provide reliable cues to stress assignment, it is unlikely that such an account would work in English. To learn the statistical relationship between a word beginning or word ending and stress assignment, the learner needs to represent the orthographic input in such a way as to identify the word beginning and word ending. This turns out to be a challenge in English. For example, when the definition of *word beginning* used by Arciuli and Cupples (2007) is applied to the whole lexicon of disyllables, many hundreds of words are exposed in which the word beginning includes the vowel of the second syllable (i.e., part of the word ending; e.g., *quiet*, *ruin*, *dial*, *react*, *triumph*, *stoic*). Similarly, there are many hundreds of words that do not have a word ending (on the definition given by Arciuli & Cupples, 2006) as a result of syllabic “l” (e.g., *apple*, *drizzle*, *bubble*), syllabic “m” (e.g., *schism*, *spasm*, *rhythm*), or falling diphthongs (e.g., *scour*, *squire*). It is also unclear how to classify the letter *Y*; this must be treated as a vowel in *abyss* but as a consonant in *beyond*. Arciuli and Cupples (2006, 2007) avoided these problems because they selected only a very small proportion of the many thousands of possible word beginnings and word endings, in which these units could be unambiguously identified. However, these challenges would need to be solved for this type of account of English reading aloud to be viable.

### Orthographic, Phonological, and Morphological Cues

In addition to these distributional cues to stress, other forms of phonological and orthographic information have been argued to provide sublexical cues to assigning stress in English polysyllables. Several researchers have argued that vowel length and the phonological weight of a syllable are important determinants of stress (Baker & Smith, 1976; Chomsky & Halle, 1968; Hayes, 1982; Kelly, 2004; Mousikou, Sadat, Lucas, & Rastle, 2017; Smith & Baker, 1976), such that syllables containing many phonemes (e.g., consonant clusters in the coda, as in col*lapse*, e*lect*) and/or long vowels (e.g., a*tone*, di*vine*) tend to attract stress in pronunciation tasks. Morphological units are also thought to provide important cues to stress assignment. Rastle and Coltheart (2000) provided evidence that prefixes (e.g., *re-*, *mis-*) repel stress when typical adults read disyllabic nonwords aloud (Rastle & Coltheart, 2000). The association between prefixes and final stress is also evident when patients with acquired surface dyslexia attempt to read disyllabic prefixed words aloud. In such cases, these patients tend to make stress errors (e.g., reading the word *reflex* with second-syllable stress; Ktori, Tree, Mousikou, Coltheart, & Rastle, 2016). Finally, it has been argued across a number of experiments that syllables with greater orthographic weight (i.e., as defined by the number of letters) and/or syllables with redundant letters tend to attract stress (e.g., the final “te” in *roulette*, Kelly, Morris, & Verrekia, 1998; Mousikou et al., 2017).

### Confounding of Cues in Empirical Work

Thus far, there is evidence that a large variety of sublexical cues may influence stress assignment in reading aloud. However, studies in this domain have typically confounded some or all of these cues. For example, Rastle and Coltheart (2000) argued that participants reliably assign second-syllable stress to nonwords such as *misbane* because of the presence of prefixes (in this case, *mis-*). However, *misbane* also has high orthographic weight and a long vowel in the second syllable, possibly biasing the reader toward second-syllable stress. Similarly, Arciuli and Cupples (2006) claimed that readers are likely to assign second-syllable stress to certain nonwords, such as *aject*, because the ending, *-ect*, is typically associated with second-syllable stress. However, this nonword also contains a prefix (i.e., *a-*) and its second syllable has high orthographic weight, thus potentially biasing the reader toward second-syllable stress. Likewise, in the Arciuli and Cupples’ (2007) preliminary study of the impact of word beginnings on stress decisions, all of the beginnings associated with second-syllable stress were also prefixes. In contrast, the beginnings that were not associated with second-syllable stress were never prefixes. Finally, Smith and Baker (1976) argued that a nonword like *gevesp* should receive more second-syllable stress than a nonword like *nodud*, because the former contains more letters in the second syllable. However, in addition to more letters, *gevesp* contains more phonemes in the second syllable than *nodud*, which could also bias stress assignment toward the second syllable. These authors also inadvertently included prefixes in some of their stimuli, which is another uncontrolled potential cue to second-syllable stress.

In a recent megastudy of disyllabic reading, Mousikou et al. (2017) sought to disentangle some of these cues. Although they found evidence for individual contributions of vowel length and orthographic weight, they were unable to determine whether prefixation contributed to stress assignment due to its confounding with other sublexical cues to stress. Consequently, we do not have a clear understanding of the individual influences of word-level cues on stress assignment. Mousikou et al. (2017) suggested that one possibility would be to conduct factorial experiments with nonwords purposely designed to pull these interrelated cues apart. Therefore, in the present work, we conducted a series of carefully-constructed reading aloud experiments (Experiments 1 through 3), in which we sought to delineate the individual influences of prefixation, orthographic weight, and vowel length on stress assignment in English. In order to strengthen our conclusions about the contribution of these sublexical cues, we also included in our analyses the potential confounding variables of word ending frequency (Sulpizio et al., 2013), the association between word endings and stress assignment (Arciuli & Cupples, 2006), lexical similarity (orthographic Levenstein distance [OLD] 20; Yarkoni, Balota, & Yap, 2008), and bigram frequency.^1^

## Sentence-Level Cues to English Stress Assignment

In addition to the sublexical cues discussed earlier, stress assignment appears to be strongly influenced by contextual factors that operate beyond the level of the single word. Most importantly, the grammatical category to which a word belongs is associated with specific stress patterns (Chomsky & Halle, 1968; Howard & Smith, 2002; Kelly & Bock, 1988; Liberman & Prince, 1977). In particular, the majority of English disyllabic nouns (approximately 90%) in CELEX take first-syllable stress, while most disyllabic verbs (approximately 67%) tend to be stressed on the second syllable (Howard & Smith, 2002). Accordingly, Arciuli and Cupples (2003) showed that grammatical class judgments are faster and more accurate for typically stressed words (i.e., nouns with first-syllable stress and verbs with second-syllable stress) than for atypically stressed words (i.e., nouns with second-syllable stress and verbs with first-syllable, stress). More recently, Breen and Clifton (2011, 2013) embedded stress-alternating noun/verb homographs in sentences (i.e., (a) The brilliant *abstract* the best ideas from the things they read; (b) The brilliant *report* the best ideas from the things they read; (c) The brilliant *abstract* was accepted at the prestigious conference; (d) The brilliant *report* was accepted at the prestigious conference). Longer reading times were found for “from the things they read” (sentences a and b) than for “at the prestigious conference” (sentences c and d), thus showing that readers had to shift their (preferred) syntactic representation of the words *abstract* and *report* from noun to verb. Interestingly, this association between grammatical class and stress has also been observed with nonwords, whose stress assignment is influenced by the syntactic context in which they are placed (Baker & Smith, 1976; Kelly & Bock, 1988; Smith & Baker, 1976; Smith, Baker, & Groat, 1982). Readers are more likely, for example, to assign first-syllable stress to a disyllabic nonword such as *pralel* when this is placed into a noun context (e.g., “The *pralel* caught the bird”) compared with when it is placed in a verb context (e.g., “The hunter *pralel* the bird”; Kelly & Bock, 1988).

Rhythm, and specifically the alternation between strong and weak beats, is another sentence-level factor that has been shown to affect stress placement in reading aloud (Kelly & Bock, 1988; Kentner, 2012). For example, in both of these sentences, “Use the *pralel* proudly” and “Planes will *pralel* pilots,” the nonword *pralel* is preceded by a weak beat and its stress is thus biased toward a strong beat (i.e., trochaic context). Conversely, in the sentences, “The proud *pralel* proposed” and “The pins *pralel* balloons,” the nonword *pralel* is preceded by a strong beat and its stress is thus biased toward a weak beat (i.e., iambic context). Kelly and Bock (1988) found that a nonword placed in a trochaic-biased context (i.e., strong–weak) was more likely to receive first-syllable stress compared with the same nonword placed in an iambic-biased context (i.e., weak–strong), irrespective of syntactic context. In a more recent study, Kentner (2012) constructed German sentences in which syntactic ambiguity could be resolved by the stress assigned to a target word, which could function either as an adverb or as a comparative quantifier. Kentner (2012) reported that during reading aloud and silent reading, participants appeared to generate an implicit prosodic representation based on the principle of rhythmic alternation of syllables (i.e., avoiding stress clashes due to adjacent stressed syllables). Critically, this implicit prosodic representation biased syntactic analysis of the target word, even though this led to integration difficulties on some trials.

The work of Kentner (2012) suggests that prosodic and syntactic cues arising at the sentence level can interact with one another. However, research is virtually silent on how these types of sentence-level cues impact on the sublexical cues typically studied in the domain of reading aloud. This is an important shortcoming, as any theory of reading that aspires to completeness must consider how factors operating at the level of single words may be influenced when words are presented in the context of whole sentences. If we step away from the specific problem of stress assignment, we are unaware of any theory that describes the mechanisms that underpin reading aloud in sentences. Further, although there is a small amount of empirical research comparing reading behavior when single words are read aloud versus read silently in sentences (Kuperman, Drieghe, Keuleers, & Brysbaert, 2013), we are unaware of any work that has compared reading aloud in single-word versus sentence-reading contexts.

In the domain of stress assignment, two studies conducted 40 years apart have begun to address this gap. The first of these studies was conducted by Baker and Smith (1976; see also Smith & Baker, 1976), who asked participants to read aloud nonwords appearing in noun or verb contexts within sentences. They observed that sublexical cues thought to operate at the word level (e.g., vowel length) were observed in these sentence contexts, suggesting that higher-level contextual cues do not completely override lower-level sublexical cues. However, there are a number of serious problems with this work, which undermine the conclusions that can be drawn. First, no data for nonwords read aloud in isolation were presented, making it difficult to ascertain the impact of grammatical category on the sublexical cues tested. Second, in addition to the confounding of different sublexical cues discussed earlier, these studies included very close analogies to existing words (e.g., zeranda, estonish, thrombossis), thus making it difficult to distinguish between potential effects of sublexical cues and word neighbors. They also included nonwords that were sufficiently difficult as to promote a “very cautious, syllable by syllable” reading strategy, which does not resemble natural reading (e.g., tupaivend, ollanteam; Baker & Smith, 1976, p. 23). Finally, the sentence contexts in which these nonwords were placed were not controlled for rhythm, as the impact of this factor on stress assignment only became apparent later through the work of Kelly and Bock (1988).

More recently, Spinelli and colleagues (Spinelli, Sulpizio, Primativo, & Burani, 2016) have returned to this issue by investigating the impact of contextual information on the effect of stress neighborhood consistency, which is frequently observed on single-word reading in Italian (see Sulpizio et al., 2015, for a review). They found that when contextual information (e.g., gender and number in the case of nouns; person in the case of verbs) is present, this information substantially overrides stress neighborhood consistency at the level of the single word. This result suggests a complex interaction between contextual cues and cues operating at the level of single words, perhaps where context contributes to the prosodic structure of a phrase, which in turn constrains the processing of individual words within the phrase. However, Spinelli et al. (2016) argued that further research is necessary to draw firm conclusions. They suggested that research in which words and nonwords are placed in sentence contexts would be particularly desirable, “as the presence of a context may affect stress processing in non-obvious ways” (Spinelli et al., 2016, p. 9)

For all of these reasons, our empirical work includes a fourth experiment, in which we investigate how the higher-level contextual cues of grammatical category and rhythm interact with the lower-level sublexical cues identified through Experiments 1, 2, and 3, to influence stress assignment in reading aloud English sentences.

## Models of Stress Assignment in English

**Figure 1 fig1:**
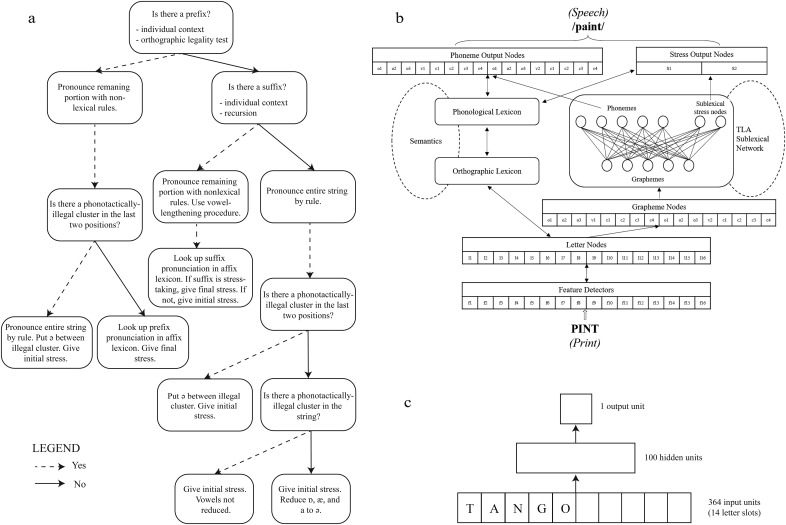
Disyllabic models of stress assignment in English: (a) The rule-based algorithm of Rastle and Coltheart (2000), (b) the CDP++ model (Perry, Ziegler, & Zorzi, 2010), and (c) the Ševa, Monaghan, and Arciuli (2009) model of stress assignment.

The problem of stress assignment has thus far been considered in three computational accounts of single-word reading in English: the rule-based algorithm proposed by Rastle and Coltheart (2000), the connectionist dual process model (CDP++; Perry et al., 2010), and the distributed-connectionist network proposed by Ševa et al. (2009). These accounts adopt different approaches to stress assignment during disyllabic word reading, and to the way sublexical cues may affect this process. These models are restricted to the processing of isolated letter strings: they have nothing to say about the impact of higher-level contextual cues that may arise in sentence reading contexts. There are, of course, many computational models of sentence reading, including models of eye-movement control (e.g., Reichle, Pollatsek, & Rayner, 2012), discourse processing (Kintsch, 1988), and syntactic parsing (e.g., Frazier & Rayner, 1982; see Rayner & Reichle, 2010; Reichle, 2015, for reviews). However, none of these models has anything to say about the computation of phonology required for reading aloud. Thus, while there is a substantial theoretical basis for understanding stress assignment at the level of the single word, there is as yet no model to offer predictions about how higher-level contextual cues may constrain processing at the level of the single word. In the following, we therefore describe the three models of single-word reading aloud presently available. The three models are shown in Figure 1.[Fig-anchor fig1]

The Rastle and Coltheart algorithm (hereafter referred to as RC00; see Figure 1a) is a partial implementation of the sublexical pathway of the DRC model (Coltheart et al., 2001), which computes the phonology of a word using a set of grapheme-to-phoneme rules. In this rule system, the spelling-to-sound conversion follows the grapheme-to-phoneme correspondence rules identified and used by the DRC model (Rastle & Coltheart, 1999; and subsequently, Coltheart et al., 2001), while stress placement is determined following the identification of orthographic strings that correspond to prefixes and suffixes. Specifically, the identification of a prefix (e.g., *pre-*, *de-*, *dis-*. *re-*, *mis-*) results in the assignment of second-syllable stress, whereas the identification of a suffix results in the assignment of first-syllable stress (except for a small group of stress-taking suffixes, such as *-een*, *-ique*, *-oo*, identified by Fudge, 1984). In the absence of an identifiable affix, the default first-syllable stress pattern of English disyllables is assigned. Evaluation of the RC00 revealed very good performance in stressing disyllabic words, as the algorithm assigned stress correctly to almost 90% of all English disyllabic words present in the CELEX database (Baayen, Piepenbrock, & van Rijn, 1993). Furthermore, when compared against human stress assignments to disyllabic nonwords, the algorithm predicted the modal human stress for 93% of items that received first-syllable stress and 75% of items that received second-syllable stress (Rastle & Coltheart, 2000).

The CDP++ model (Perry et al., 2010; see Figure 1b) is a dual-pathway model of disyllabic reading aloud that is built on its predecessor, the CDP+ model of monosyllabic reading aloud (Perry et al., 2007). Unlike the RC00 algorithm, the CDP++ model is a full processing model that produces a pronunciation, stress marker, and reaction time (RT). The CDP++ is very similar to the CDP+ model except for an increase in the number of letter and phoneme slots to accommodate longer words, an expanded input coding template to accommodate disyllables, the introduction of the schwa phoneme to deal with vowel reduction, the inclusion of stress nodes to represent the position of stress, and the use of a larger training corpus and lexicon. The lexical procedure of the CDP++ model is identical to that of the DRC model, storing item-based knowledge about the orthography and phonology of a known word. Accordingly, the lexical procedure of the CDP++ model directly activates the stress that is associated with a familiar word’s spoken form. The sublexical procedure of this model incorporates a two-layer associative (TLA) network for mapping graphemes onto phonemes, unlike the set of rules used by the DRC model, and by extension, the RC00 algorithm. In the TLA network, the orthographic input is organized along a graphosyllabic structure, which segments each syllable into onset (i.e., the initial consonant graphemes) and rime (i.e., the vowel and any following consonant graphemes) units that make direct contact with the corresponding phonological units and two sublexical stress units. During a training phase, the TLA network uses a connectionist algorithm to learn grapheme-to-phoneme and grapheme-to-stress associations based on statistical regularities across the model’s training set. Accordingly, the sublexical procedure of the CDP++ model activates the stress pattern that it learnt to associate with certain graphemes in order to assign stress to unfamiliar letter strings. The performance of the CDP++ model on stressing disyllabic words was evaluated against several databases (English Lexicon Project database; Balota et al., 2007; Chateau & Jared, 2003; Yap & Balota, 2009) and in all cases, the model was highly successful in predicting the correct stress pattern of words, with the majority of stress errors resulting from the model assigning first-syllable stress to words with second-syllable stress. The CDP++ model’s performance was also assessed against the human nonword reading aloud data of Rastle and Coltheart (2000). The model predicted the modal human stress for approximately 92% of items that received first-syllable stress and 51% of items that received second-syllable stress.

The Ševa et al. (2009) network (hereafter referred to as SMA09; see Figure 1c), also uses a distributed-connectionist framework to simulate stress placement in English disyllables during reading aloud, but provides no pronunciation or RT. This model uses the statistical regularities available in its training set to learn how to map an orthographic input onto a stress pattern but it differs from the CDP++ in three key aspects. First, though the CDP++ model uses a structured template representing onsets and rimes in each syllable, the SMA09 model organizes its orthographic input using a simple left-aligned, slot-based coding scheme. Second, though the orthographic and phonological representations are directly connected in the CDP++, the SMA09 includes an additional intermediate layer of hidden units between its orthographic input and the phonological output layers, which could potentially contribute to learning more complex relationships between orthography and phonology. Finally, the training set of the SMA09 model includes only disyllabic words, whereas the CDP++ is trained on both monosyllabic and disyllabic words. The SMA09 model’s performance on assigning stress to a subset of disyllabic words drawn from the CELEX database proved to be slightly better than the RC00 algorithm (87% and 84% of correct stress predictions, respectively). However, the model performed less well than the RC00 algorithm and the CDP++ model in predicting the human modal stress assigned to the group of disyllabic nonwords used in the Rastle and Coltheart (2000) study. Specifically, the SMA09 model predicted the correct modal stress pattern for almost 88% of the first-syllable stressed items and 50% of the second-syllable stressed items. Similarly to the CDP++ model, the SMA09 network’s inferior performance was due to assigning first-syllable stress to nonwords that were given second-syllable stress by the majority of human readers. Ševa et al. (2009) argued, however, that the superior performance of the RC00 algorithm on this set of nonwords could have been due to an overrepresentation of items containing affixes, which were readily identified by the RC00 algorithm.

In summary, despite their different approaches, all three models described appear relatively successful in simulating the assignment of stress in English disyllables. Further, they account for the role of sublexical cues on stress assignment from different standpoints. On one hand, the RC00 algorithm employs a set of all-or-none rules that are governed by the morphological structure of a word. On the other hand, the CDP++ model and the SMA09 network adopt a statistical learning approach, which allows for the discovery of print-to-stress regularities in the language. In the present study, we compare simulation results from these models against data from Experiment 1 through 3 (isolated presentation) to examine which of these alternative approaches to stress assignment best captures human reading aloud performance. Though these models are unable to simulate higher-level contextual factors on stress assignment (e.g., grammatical category, rhythm), we also assess how well they predict the impact of lower-level cues when nonwords are placed in sentences (Experiment 4).

## Experiments 1 Through 3

Experiments 1 through 3 aimed to establish the effects of three sublexical cues on stress assignment during reading aloud, namely, prefixation, vowel length, and orthographic weight. In all three experiments, we asked participants to read aloud a series of carefully-constructed disyllabic nonwords, in which we systematically varied the presence of these cues and examined their effects on second-syllable stress. In Experiment 1, we examined the effect of prefixation and vowel length by presenting participants with four sets of nonwords varied factorially on the inclusion of a prefix and the length of the second vowel (e.g., *prelel*, *pralel*, *preleal*, *praleal*). In Experiment 2, we examined the effects of prefixation and orthographic weight by presenting participants with four sets of nonwords varied factorially on the inclusion of a prefix and the number of letters contained in the second syllable (e.g., *prelel*, *pralel*, *prelell*, *pralell*). In Experiment 3, we reexamined the effects of prefixation and vowel length while controlling for orthographic weight. We achieved this by presenting participants with four sets of nonwords that contained the same number of letters in the second syllable and were varied factorially on the inclusion of a prefix and the length of the second vowel (e.g., *prelell*, *pralell*, *preleal*, *praleal*). This experimental approach allowed us to ascertain the influence of each one of these sublexical cues, independently and in combination with another cue, on the assignment of second-syllable stress.

### Experiment 1: Prefixation and Vowel Length

Experiment 1 examined the effects of prefixation and vowel length on stress assignment during nonword reading aloud. We predicted that readers would be more likely to assign second-syllable stress to prefixed nonwords compared with non-prefixed nonwords, and to nonwords with a long vowel in their second syllable compared with nonwords with a short vowel in their second syllable.

#### Participants

Twenty undergraduate students from Royal Holloway, University of London, were paid £5 to participate in the study. Participants were monolingual native speakers of Southern British English and reported no visual, reading, or language difficulties.

#### Stimuli and design

Stimuli comprised 80 phonotactically legal nonwords, ranging in length from five to seven letters. All nonwords were disyllabic with at least one medial consonant flanked by two vowels. Nonwords were varied factorially on (a) the inclusion of a prefix and (b) the length of the second vowel, thus yielding 20 prefixed nonwords with a short vowel in the second syllable (prefixed short vowel), 20 prefixed nonwords with a long vowel in the second syllable (prefixed long vowel), 20 non-prefixed nonwords with a short vowel in the second syllable (non-prefixed short vowel), and 20 non-prefixed nonwords with a long vowel in the second syllable (non-prefixed long vowel).

Nonword construction was performed in such a way so that items were pairwise matched between the different conditions, with prefixes (*de-*, *mis-*, *pre-*, *re-*) changing into non-prefixes (*do-*, *mes-*, *pra-*, *ro-*) and short vowels (a, e, o, u) changing into long vowels (ai, ea/ee, oa, oo/ou) in a consistent manner. For example, nonwords beginning with the prefix *pre-* in the prefixed conditions would be matched to nonwords beginning with the letter sequence *pra-* in the non-prefixed conditions (e.g., the prefixed item *prelel* was matched to the non-prefixed item *pralel*). Likewise, nonwords containing the short vowel “e” as a second vowel in the short-vowel conditions would be matched to nonwords containing the long vowel “ea” or “ee” as a second vowel in the long-vowel conditions (e.g., the items *prelel* and *pralel* were matched to the items *preleal* and *praleal*, respectively). This pairwise item matching was undertaken to ensure that differences across conditions were minimized apart from the experimental manipulations of interest. Also, stimuli were constructed in such a way that prefixes and their control orthographic counterpart units were likely to overlap with the first syllable of the nonword (e.g., in nonwords such as *misdut* and *mesdut*, the letter sequence “sd” results in a phonotactically illegal second-syllable onset, hence these nonwords would be most likely syllabified as *mis.dut* and *mes.dut*, respectively). Finally, care was taken to ensure that neither of the two syllables of the nonwords contained a monosyllabic English word.

Nonwords in the four conditions were group-wised matched on orthographic neighborhood size (Coltheart’s N; Coltheart, Davelaar, Jonasson, & Besner, 1977): prefixed short vowel (*M* = 0.15, *SD* = 0.37); prefixed long vowel (*M* = 0, *SD* = 0); non-prefixed short vowel (*M* = 0.10, *SD* = 0.31); non-prefixed long vowel (*M* = 0.05, *SD* = 0.22); *F*(1, 76) < 2.87, *p* > .05). Further, nonwords across the four conditions did not differ in terms of the number of their orthographic neighbors that take second-syllable stress, *F*(1, 76) < 2.11, *p* > .05.

Nonwords were further divided into four blocks of 20 experimental trials, so that all experimental conditions were equally represented in each block but no pairwise-matched nonwords appeared in the same block. Nonwords in each block were presented in a random order, while the order of presentation of the blocks was counterbalanced across participants. Following a practice session of five trials, each participant was presented the four blocks. The nonword stimuli used in Experiment 1 are listed in Appendix A.

#### Apparatus and procedure

Participants were tested individually in a quiet room. Each participant sat approximately 40 cm in front of a CRT monitor. Stimulus presentation and data recording were controlled by DMDX software (Forster & Forster, 2003), and verbal responses were recorded by a head-worn microphone. Nonword stimuli were displayed in white on a black background in a 14-point Courier New font. Each trial began with the presentation of a fixation cross in the center of the screen for 500 ms. The fixation cross was replaced at the same location with a nonword stimulus, which was displayed for 3,000 ms. Participants were asked to read aloud the nonword into the microphone as quickly and as clearly as possible, as if it were a real word. The next trial followed after a 850-ms blank interval.

#### Results

The analyses included responses to a total of 76 items per participant (due to an oversight, a group of four pairwise-matched stimuli was not presented to the participants). Nonword responses were excluded if they were pronounced with anything other than two syllables, or if they were characterized by hesitations or other articulatory dysfluencies (1.8% of the data). The remaining responses were classified as being stressed on the first or second syllable. Stress judgments were undertaken by one of the authors (K.R.) who had previous training and experience in such a task (Mousikou et al., 2017; Rastle & Coltheart, 2000).^2^ The proportions of second-syllable stress in the four different conditions are presented in Table 1.[Table-anchor tbl1]

In this and all following experiments, we analyzed the impact of stimulus factors on stress assignment (a binary variable) using generalized linear mixed-effects models. These analyses were performed using the packages lme4 (Bates, Maechler, Bolker, & Walker, 2015), car (Fox & Weisberg, 2011), multcomp (Hothorn, Bretz, & Westfall, 2008), and lsmeans (Lenth, 2016) implemented in the statistical software R (Version 3.3.1; R Core Team, 2016). In each experiment, a logit mixed model (Jaeger, 2008) was created using stepwise backward model comparison and model selection was performed on the basis of chi-squared log-likelihood ratio tests. The significance of the fixed effects was determined with Type III model comparisons using the Anova function provided by the car package.

Nonword items were matched across conditions on orthographic neighborhood size (Coltheart’s N). However, many of our items had neighborhoods of zero on this measure. Hence, we included two more sensitive measures of orthographic similarity, namely the OLD20 (Yarkoni et al., 2008) and mean bigram type frequency, as covariates in the analyses. Furthermore, given the evidence that the endings of words can carry orthographic cues to stress assignment in English (Arciuli & Cupples, 2006), we included two additional covariates in our analyses, the total number of disyllabic words in CELEX that share the same endings as our stimuli (ending frequency) and the proportion of these words that take second-syllable stress (ending-to-second-syllable-stress proportion). Following Arciuli and Cupples (2006), *word ending* was defined as the orthographic unit that includes all the final letters of a word starting from the nucleus of the second syllable (e.g., -*ow* in *follow*, *-ark* in *embark*, and *-upt* in *erupt*). These steps were taken to ensure that any differences between our conditions of interest would not be due to the frequency of occurrence of the items’ sublexical orthographic units in the lexicon, or to the association of these units with second-syllable stress in English disyllabic words. However, in the following analyses, we focus only on the results pertaining to our factors of interest.

Our model included stressed syllable (first vs. second) as the dependent variable, prefixation (non-prefixed vs. prefixed), second-vowel length (short vs. long) and their interaction as fixed effects, and participants and items as crossed random effects. OLD20, χ^2^(1) = 7.36, *p* = .007, and ending-to-second-syllable-stress proportion, χ^2^(1) = 12.83, *p* < .001, were the only covariates that contributed significantly to the model’s goodness of fit and were thus included in the final model.

Results revealed a main effect of prefixation, χ^2^(1) = 16.78, *p* < .001, with prefixed nonwords receiving more second-syllable stress than non-prefixed nonwords. As predicted by the fitted logistic regression model, the probability of second-syllable stress for non-prefixed items was 28%, whereas the corresponding probability for prefixed items was 45%. There was also a main effect of second-vowel length, χ^2^(1) = 32.00, *p* < .001, with nonwords containing a long second vowel receiving more second-syllable stress than those with a short second vowel. The predicted probability of second-syllable stress for items with a short second vowel was 23%, whereas the corresponding probability for items with a long second vowel was 52%. The interaction between prefixation and vowel length was not significant, χ^2^(1) = 0.17, *p* = .679.

## Discussion

Results revealed that both prefixation and vowel length influenced the assignment of stress in nonword reading aloud. Readers were more likely to assign second-syllable stress to disyllabic nonwords that contained a prefix compared with non-prefixed nonwords. Similarly, readers were more likely to assign second-syllable stress to disyllabic nonwords with a long second vowel compared with nonwords with a short second vowel. The lack of a significant interaction between prefixation and vowel length suggests that these sublexical cues constitute independent sources for predicting the assignment of stress. The additive effects of prefixation and vowel length can be clearly seen in the prefixed long vowel condition, where prefixed nonwords with a long second vowel (e.g., *preleal*) received the highest proportion of second-syllable stress.

In this experiment, however, the influence of vowel length was confounded with the potential effect of another sublexical cue, that is, the orthographic weight of a syllable. This is because nonwords with a long second vowel (e.g., *praleal*, *preleal*) also contained more letters in their second syllable compared to nonwords with a short second vowel (e.g., *pralel*, *prelel*), as is typical in English spelling-to-sound mappings. Therefore, it is possible that the observed effect of vowel length is driven by the combination of vowel length and orthographic weight, or that it simply reflects a pure effect of orthographic weight, with syllables containing more letters being more likely to attract stress. Following the same factorial design, Experiment 2 was designed to establish whether the orthographic weight of a syllable influences the assignment of stress and whether it interacts with the observed effect of prefixation.

### Experiment 2: Prefixation and Orthographic Weight

Experiment 2 examined the effects of prefixation and the orthographic weight (i.e., number of letters) of the second syllable on stress assignment during nonword reading aloud. To avoid confounds with potential effects of vowel length on stress assignment (as those observed in Experiment 1), all nonword stimuli contained a short vowel. We predicted that readers would be more likely to assign second-syllable stress to prefixed nonwords compared with non-prefixed nonwords, and to nonwords that contain more letters in their second syllable compared with nonwords that contain fewer letters in their second syllable.

#### Participants

Twenty newly recruited undergraduate students from Royal Holloway, University of London, were paid £5 to participate in the study. Participants were monolingual native speakers of Southern British English and reported no visual, reading, or language difficulties.

#### Stimuli and design

Stimuli comprised 80 phonotactically legal disyllabic nonwords, which ranged in length from five to seven letters. Nonwords were varied factorially on (a) the inclusion of a prefix and (b) the orthographic weight of the second syllable (operationalized as containing three or four letters), yielding 20 prefixed nonwords with a short second syllable (prefixed light syllable), 20 prefixed nonwords with a long second syllable (prefixed heavy syllable), 20 non-prefixed nonwords with a short second syllable (non-prefixed light syllable), and 20 non-prefixed nonwords with a long second syllable (non-prefixed heavy syllable). Nonwords with a light second syllable were the same stimuli as those used in the prefixed and non-prefixed short vowel conditions in Experiment 1. Nonword stimuli with a heavy second syllable were pairwise matched to those with a light second syllable, and were constructed by adding an additional consonant letter to their second syllable. Specifically, added letters that represent a single phoneme were placed either at the beginning or at the end of the second syllable to form a digraph such as “ph,” “sh,” and “th,” or a double consonant cluster such as “ng,” “ss,” and “ll.” For example, the item *prelel* in the prefixed light syllable condition was matched to the item *prelell* in the prefixed heavy syllable condition, the item *pralel* in the non-prefixed light syllable condition, and the item *pralell* in the non-prefixed heavy syllable condition. Thus, all nonwords had the same number of phonemes and consisted of a short vowel in their second syllable. As with Experiment 1, care was taken so that prefixes and their control orthographic counterpart units comprised the first syllable of the nonword. Further, neither of the two syllables of the nonwords corresponded to an English monosyllabic word.

Nonwords in the four conditions were group-wise matched on Coltheart’s N: prefixed light syllable (*M* = 0.15, *SD* = 0.37); prefixed heavy syllable (*M* = 0, *SD* = 0); non-prefixed light syllable (*M* = 0.10, *SD* = 0.31); non-prefixed heavy syllable (*M* = 0.05, *SD* = 0.22); *F*(1, 76) < 2.87, *p* > .05. Further, nonwords in the four conditions did not differ in terms of the number of their orthographic neighbors that take second-syllable stress, *F*(1, 76) < 2.11, *p* > .05.

As in Experiment 1, nonwords were divided into four blocks of 20 items, so that all experimental conditions were equally represented but no pairwise-matched nonwords appeared in the same block. Nonwords in each block were presented in a random order, whereas the presentation order of each block was counterbalanced across participants. Following a practice session of five trials, each participant was presented the four blocks. The nonword stimuli used in Experiment 2 are listed in Appendix B.

#### Apparatus and procedure

The apparatus and procedure were the same as in Experiment 1.

#### Results

Nonword responses were classified as having stress on the first or second syllable, while hesitations and responses with anything other than two syllables were marked as erroneous and discarded (1.1% of all responses). Table 2 displays the proportion of second-syllable stress across the four conditions.[Table-anchor tbl2]

As for Experiment 1, stress assignment data were analyzed using a generalized linear mixed-effects model. Our model included stressed syllable (first vs. second) as the dependent variable, prefixation (non-prefixed vs. prefixed), orthographic weight of the second syllable (light vs. heavy) and their interaction as fixed effects, and participants and items as crossed random effects. OLD20, χ^2^(1) = 8.55, *p* = .003, and mean bigram frequency, χ^2^(1) = 19.19, *p* < .001, contributed significantly to the model’s goodness of fit and were thus included in the final model as covariates.

Results revealed a main effect of prefixation, χ^2^(1) = 15.64, *p* < .001, with prefixed nonwords receiving more second-syllable stress than non-prefixed nonwords. As predicted by the fitted logistic regression model, the probability of second-syllable stress for non-prefixed items was 10%, whereas the corresponding probability for prefixed items was 22%. There was also a main effect of orthographic weight, χ^2^(1) = 8.46, *p* = .004, with nonwords containing a heavy second syllable receiving more second-syllable stress than those with a light second syllable. The predicted probability of second-syllable stress for items with a light second syllable was 10%, whereas the corresponding probability for items with a heavy second syllable was 22%. The interaction between prefixation and orthographic weight was not significant, χ^2^(1) = 0.55, *p* = .460.

Correlational analyses examined the consistency with which participants assigned stress to the subsets of nonword items that appeared both in Experiments 1 and 2 (i.e., the nonwords in the prefixed and non-prefixed short vowel conditions of Experiment 1 and the nonwords in the prefixed and non-prefixed light syllable conditions of Experiment 2). Results established high item-based reliability (*r* = .73, *p* < .001) on the assignment of stress for these nonwords across the two experiments.

## Discussion

Our results from Experiment 2 revealed an effect of prefixation on the assignment of stress in nonword reading aloud. Readers were more likely to assign second-syllable stress to disyllabic nonwords that contained a prefix than to non-prefixed nonwords, replicating the results of Experiment 1. The results from Experiment 2 also showed an effect of orthographic weight on stress assignment, as readers were more likely to assign second-syllable stress to disyllabic nonwords that contained a higher number of letters in their second syllable compared with nonwords that contained fewer letters in their second syllable. The effects of prefixation and orthographic weight were independent, as evidenced by the lack of a significant interaction between these two sublexical cues. These results confirm that orthographic weight is an independent cue to stress assignment. In Experiment 3 we sought to determine whether this was also the case for vowel length.

### Experiment 3: Prefixation and Vowel Length With Orthographic Weight Controlled

Experiment 3 reexamined the effects of prefixation and vowel length of the second syllable on stress assignment during nonword reading aloud, after controlling for the effect of orthographic weight of the second syllable. In this experiment, all nonword stimuli contained the same number of letters in their second syllable. We predicted that readers would be more likely to assign second-syllable stress to prefixed nonwords compared with nonprefixed nonwords, and to nonwords with a long vowel in their second syllable compared with nonwords with a short vowel in their second syllable.

#### Participants

Twenty newly recruited undergraduate students from Royal Holloway, University of London, were paid £5 to participate in the study. Participants were monolingual native speakers of Southern British English and reported no visual, reading, or language difficulties.

#### Stimuli and design

Stimuli comprised 80 disyllabic nonwords, ranging in length from six to seven letters. All nonwords had the same number of letters (and phonemes) in their second syllable and were varied factorially on (a) the inclusion of a prefix and (b) the length of the second vowel. Nonwords with a short vowel in the second syllable (prefixed short vowel and non-prefixed short vowel) were the same as those used in Experiment 2 in the prefixed heavy syllable condition (e.g., *prelell*) and the non-prefixed heavy syllable condition (e.g., *pralell*), respectively. Nonwords with a long vowel (prefixed long vowel and non-prefixed long vowel) were the same as those used in Experiment 1 in the prefixed long vowel condition (e.g., *preleal*) and the non-prefixed long vowel condition (e.g., *praleal*), respectively. Because of the way nonwords were constructed in Experiments 1 and 2, items were already pairwise matched between the different conditions of Experiment 3.

Nonwords across the four conditions were group-wise matched on Coltheart’s N: prefixed short vowel (*M* = 0, *SD* = 0); prefixed long vowel (*M* = 0, *SD* = 0); non-prefixed short vowel (*M* = 0.05, *SD* = 0.22); non-prefixed long vowel (*M* = 0.05, *SD* = 0.22); *F*(1, 76) < 2.00, *p* > .05. None of the nonwords had orthographic neighbors that take second-syllable stress.

Following the same rationale as in Experiments 1 and 2, nonwords were divided into four blocks of 20 items. All experimental conditions were equally represented but no pairwise-matched nonwords appeared in the same block. Nonwords in each block were presented in a random order, while the presentation order of each block was counterbalanced across participants. Following a practice session of five trials, each participant was presented the four blocks. The nonword stimuli used in Experiment 3 are listed in Appendix C.

#### Apparatus and procedure

The apparatus and procedure were the same as in Experiments 1 and 2.

#### Results

Disyllabic responses were classified as having received stress on the first or the second syllable, while hesitations and responses with anything other than two syllables were marked as erroneous and discarded (2.4% of all responses). Table 3 displays the proportion of second-syllable stress responses across the four conditions.[Table-anchor tbl3]

As for Experiments 1 and 2, stress assignment data were analyzed using generalized linear mixed-effects model. Our model included stressed syllable (first vs. second) as the dependent variable, prefixation (non-prefixed vs. prefixed), second-vowel length (short vs. long) and their interaction as fixed effects, and participants and items as crossed random effects. OLD20, χ^2^(1) = 15.71, *p* < .001, contributed significantly to the model’s goodness of fit and was thus included in the final model as a covariate.

Results revealed a main effect of prefixation, χ^2^(1) = 11.90, *p* < .001, with prefixed nonwords receiving more second-syllable stress than non-prefixed nonwords. As predicted by the fitted logistic regression model, the probability of second-syllable stress for non-prefixed items was 30%, whereas the corresponding probability for prefixed items was 52%. There was also a main effect of second-vowel length, χ^2^(1) = 6.63, *p* = .010, with nonwords containing a long second vowel receiving more second-syllable stress than those with a short vowel in the second syllable. The predicted probability of second-syllable stress for items with a short second vowel was 33%, whereas the corresponding probability for items with a long second vowel was 48%. The interaction between prefixation and vowel length was not significant, χ^2^(1) = 0.57, *p* = .449.

Correlational analyses examined stress consistency between the subsets of nonwords that appeared both in Experiments 1 and 3 (i.e., the nonwords in the prefixed and non-prefixed long vowel conditions of Experiments 1 and 3) and between the subsets of nonwords that appeared both in Experiments 2 and 3 (i.e., the nonwords in the prefixed and non-prefixed heavy syllable conditions of Experiment 2 and the nonwords in the prefixed and non-prefixed short vowel conditions of Experiment 3). Results revealed a high degree of item-based reliability (*r* = .73, *p* < .001 and *r* = .78, *p* < .001, respectively) on the assignment of stress for these nonwords across the three experiments.

## Discussion

The results from Experiment 3 replicated the findings from Experiments 1 and 2 by revealing an effect of prefixation on the assignment of stress. Readers were more likely to assign second-syllable stress to prefixed nonwords than non-prefixed nonwords. The results from the present experiment also established an effect of vowel length on stress assignment. Readers were more likely to assign second-syllable stress to nonwords with a long vowel in the second syllable than nonwords with a short second vowel. The effect of vowel length was independent of the effect of prefixation, as evidenced by the lack of a significant interaction between these two sublexical cues. Furthermore, in the current experiment, the effect of vowel length remained significant even after controlling for the orthographic weight of the second syllable, which was found to influence second-syllable stress assignment in Experiment 2. This finding provides clear evidence that the effect of vowel length is independent of the number of letters present in a syllable, and that vowel length constitutes a sublexical cue to stress in its own right.

## Model Simulations

Using the nonword stimuli presented in Experiments 1, 2, and 3, we sought to assess the performance of the RC00 algorithm (Rastle & Coltheart, 2000), the CDP++ model (Perry et al., 2010), and the SMA09 network (Ševa et al., 2009). First, in order to obtain a general index of the models’ stress assignment performance in relation to that of the human readers, we assessed the success of each model in capturing the modal stress assigned to each of the nonwords presented in Experiments 1, 2, and 3 across experiments and participants. Second, in order to determine whether the rule-based or the connectionist approach best captures the specific sensitivities that human readers show to the sublexical stress cues under investigation, we compared the human stress data obtained separately from Experiments 1, 2, and 3 with simulation results from these three accounts of disyllabic reading in English. The identification of a prefix by the RC00 algorithm unambiguously results in second-syllable stress. Hence we predicted that this model would be more likely to assign second-syllable stress to prefixed nonwords compared with non-prefixed nonwords. However, this algorithm contains no explicit rules regarding the sublexical cues of orthographic weight and vowel length, and so we did not expect it to be sensitive to these cues. The CDP++ model and the SMA09 network adopt a statistical-learning approach that does not allow the explicit formulation of hypotheses in respect of the sublexical cues under investigation. Hence, we made no predictions about the performance of these models.

## Model Performance in Capturing Modal Human Stress Assignment

We calculated the modal stress produced by human participants for each of the 120 items that were presented across Experiments 1, 2, and 3. Participants assigned first-syllable stress to 75% of the items and second-syllable stress to 25% of the items, mirroring the distribution of stress in English disyllables (Baayen et al., 1995). To test the models’ performance against the modal human stress, we performed a binary classification analysis. Adopting the same approach as Ševa et al. (2009), we assessed each model’s sensitivity in assigning first and second-syllable stress by using the *d′* measure, which is calculated by taking into account both the model’s correct classifications as well as its misclassifications. We further obtained a measure of response bias (*c*), indicating whether a model was biased toward first- or second-syllable stress assignment. An increase in the absolute value of *d′* and *c* coefficients would indicate an increase in the model’s stress assignment sensitivity and response bias, respectively, with a response bias toward first-syllable stress being denoted by a negative value. The distribution of stress pattern assigned by human participants and the stress pattern predicted by the models are reported in Table 4.[Table-anchor tbl4]

The RC00 algorithm predicted the modal human stress for 60% of the items that were assigned first-syllable stress and for 77% of the items that were assigned second-syllable stress (*d′* = 0.98), and revealed a response bias toward second-syllable stress (*c* = .24). The CDP++ model correctly stressed 88% and 53% of the items that received first-syllable and second-syllable stress, respectively, by the majority of human participants (*d′* = 1.25), and showed a response bias toward first-syllable stress (*c* = −.54). Finally, the SMA09 network predicted first and second-syllable stress for 64% and 53% of the items (*d′* = 0.45), respectively, in accordance with the modal human stress, and showed a small response bias toward first-syllable stress (*c* = −.14). This analysis thus suggests that the CDP++ model performed slightly better than the other two models in predicting human stress assignment.

## Model Sensitivity to Sublexical Cues

To ascertain whether the models were sensitive to the same sublexical cues as human readers, the simulation data for each model were submitted to logistic regression analyses. These analyses assessed the probability of second-syllable stress (a binary variable) occurring as a function of the binary variables of prefixation and second-vowel length (Experiments 1 and 3), and the binary variables of prefixation and orthographic weight (Experiment 2). The results from the logistic regression analyses are reported in Table 5.^3^

### Experiment 1

Table 1 displays the proportion of second-syllable stress as a function of prefixation and vowel length for each of the three models under consideration. For the RC00 algorithm, the simulation results revealed only a significant effect of prefixation on the assignment of second-syllable stress. As predicted by the fitted regression model, the probability of second-syllable stress in the RC00 algorithm was 3% for non-prefixed items and 98% for prefixed items. For the CDP++ model, there was only a significant effect of second-vowel length. The predicted probability of second-syllable stress in the CDP++ model was 5% for items with a short second vowel and 58% for items with a long second vowel. Finally, for the SMA09, the simulation results showed a significant effect of prefixation only. The predicted probability of second-syllable stress in the SMA09 network was 18% for nonprefixed items and 65% for prefixed items. For all three models, the interaction between prefixation and second-vowel length was not significant.

### Experiment 2

Table 2 contains the mean proportion of second-syllable stress as a function of prefixation and orthographic weight for the three models under consideration. For the RC00 algorithm, the simulation results revealed a complete separation for the effect of prefixation; whether an item contained a prefix completely determined the likelihood of second-syllable stress in the model. Neither prefixation nor orthographic weight influenced the proportion of second-syllable stress in the CDP++ model. Finally, for the SMA09 network, there was a significant effect of prefixation. The predicted probability of second-syllable stress in the SMA09 network was 13% for non-prefixed items and 58% for prefixed items. For all three models, the effect of orthographic weight and the interaction between prefixation and orthographic weight were not significant.

### Experiment 3

Table 3 displays the mean proportion of second-syllable stress as a function of prefixation and vowel length for the three models under consideration. For the RC00 algorithm and the CDP++ model, simulation results were identical to those of Experiment 1. The RC00 revealed a significant effect of prefixation, and predicted second-syllable stress for 3% of non-prefixed items and for 97% of prefixed items. For the CDP++ model, simulation results revealed a significant effect of vowel length. The predicted probability of second-syllable stress in the CDP++ model was 5% for items with a short second vowel and 58% for items with a long second vowel. For the SMA09 network, there was a significant effect of prefixation. The predicted probability of second-syllable stress in the SMA09 network was 20% for non-prefixed items and 68% for prefixed items. For all three models, the interaction between prefixation and second-vowel length was not significant.

## Discussion

The general performance of the models in capturing the human modal stress on the complete set of our experimental stimuli varied. The CDP++ model was the most successful in predicting human stress overall, but despite its good performance in predicting first-syllable stress, its performance in predicting second-syllable stress was less impressive. The RC00 algorithm performed less well and revealed a substantial bias toward second-syllable stress assignment. However, given that half of the experimental stimuli contained a prefix, and that the RC00 contains a hard-wired rule that assigns second-syllable stress to any item in which a prefix is identified, the model’s second-syllable stress bias is not surprising. The SMA09 network was the least successful model in predicting human modal stress assignment.

However, the models presented a different picture when their specific sensitivity to each of the sublexical cues investigated in Experiments 1, 2, and 3 was assessed. It is clear from these simulation results that none of the three models under examination perfectly matched the performance of human readers. The RC00 algorithm showed strong sensitivity to prefixation, with prefixed nonwords being much more likely to receive second-syllable stress than non-prefixed nonwords across all three experiments (e.g., *prelel*, *prelell*, *preleal* vs. *pralel*, *pralell*, *praleal*, respectively). This finding was entirely expected, given the algorithm’s hard-wired rule regarding prefixes. However, the magnitude of the prefixation effect in RC00 far eclipsed that shown by human readers. As it is evident in Tables 1, 2, and 3, the algorithm systematically overestimated the effect of prefixation by assigning second-syllable stress to the vast majority of prefixed items (97% of all prefixed items averaged across Experiments 1, 2, and 3), whereas it failed to assign second-syllable stress to any of the items that did not contain a prefix (with the exception of a single item in Experiments 1 and 3). Further, the RC00 failed to show any sensitivity to either vowel length or orthographic weight of the syllable, suggesting that the sublexical rules contained in this model are not sufficient to capture other nonmorphological cues to stress assignment.

The CDP++ model showed sensitivity to vowel length, as nonwords that contained a long vowel in their second syllable were more likely to receive second-syllable stress than nonwords that contained a short vowel in their second syllable (the possible source of this sensitivity is discussed in the General Discussion). The CDP++ model also showed a small, yet nonsignificant effect of prefixation (see Tables 1 through 3; note that the failure to reach statistical significance should be taken in the context that the model is being treated as a single subject). Finally, the model showed no evidence of sensitivity to orthographic weight.

Interestingly, in contrast to its mediocre performance in predicting human modal stress, the SMA09 network performed considerably better than the other two models when its sensitivity to each of the sublexical cues to stress assignment was assessed. Across all three experimental manipulations, the model showed sensitivity to prefixation, suggesting that the model was able to learn an association between frequently occurring orthographic units in the beginning of a word (i.e., prefixes) and their stress pattern. It is noteworthy, however, that the magnitude of the prefixation effect predicted by the model was greater than that shown by human participants. In comparison to human readers, the model underestimated the incidence of second-syllable stress in non-prefixed items and overestimated the incidence of second-syllable stress in prefixed items (see Tables 1 through 3 and the General Discussion for a possible explanation for this effect). The model also showed subtle effects of orthographic weight and vowel length in the same direction as human readers, although these effects did not reach statistical significance in the model.

## Experiment 4

### Sublexical Cues Versus Syntactic and Rhythmic Contexts

Experiments 1, 2, and 3 provided clear evidence for the influence of prefixation, vowel length, and the orthographic weight of a syllable on the assignment of stress in single nonword reading. In this final experiment, we investigated the extent to which the effects of these sublexical cues to stress assignment interact with higher-level contextual factors that arise in sentence reading. The relationship between word-level and sentence-level cues to stress assignment is poorly understood. Some recent evidence suggests that in Italian, a sublexical cue based on distributional information about the word endings (i.e., stress neighborhood consistency) is overridden by the presence of contextual cues derived from distributional information pertaining to morpho-syntactic properties (i.e., gender, number, person; Spinelli et al., 2016). In contrast, existing evidence appears to suggest that sublexical cues in English continue to influence stress assignment in sentence reading, even in the presence of higher-level contextual factors (Baker & Smith, 1976; Smith & Baker, 1976). However, in these investigations, the influence of sublexical cues was never examined separately from the sentence context in which the nonwords were placed. Furthermore, while these studies examined the relationship between sublexical cues and syntactic context, they did not control for rhythm, which was subsequently shown to influence stress assignment in reading aloud (Kelly & Bock, 1988).

In Experiment 4, we compared stress assignment to nonwords that contained all of the three previously examined sublexical cues (thus effecting a strong bias toward second-syllable stress; e.g., preleal) with nonwords that did not contain any such cues (e.g., pralel). These nonwords were placed in sentences that were constructed to bias stress toward the first or second syllable, based on syntactic (i.e., nouns and verbs) and rhythmic (i.e., trochaic and iambic) cues. Based on Kelly and Bock’s (1988) findings, we expected that the syntactic and rhythmic cues would affect the placement of stress. Specifically, we predicted that nonwords embedded in a verb or an iambic context would receive significantly more second-syllable stress than nonwords embedded in a noun or a trochaic context, respectively.

We did not have a good prediction as to how these higher-level cues may interact with the lower-level sublexical cues. If the effects of sublexical cues are maintained in the face of the contextual factors present in sentence reading (as the work of Baker & Smith, 1976, and Smith & Baker, 1976, suggests), then we would expect to observe an influence of sublexical cues on stress assignment, so that cued nonwords should receive significantly more second-syllable stress than nonwords without any sublexical cues. On the other hand, the recent work of Spinelli et al. (2016) suggests that we may observe an interaction between higher-level and lower-level cues. One possibility is that the influence of the higher-level contextual cues will be so powerful as to minimize (or even extinguish) the impact of lower-level sublexical cues. Conversely, because the cued nonwords in the present experiment contained a combination of all three previously examined sublexical cues (and were thus heavily biased toward second syllable stress), it is also possible that these word-level cues could modulate (or even extinguish) the effects of either or both of the higher-level contextual factors. If this were the case, then we should observe a greater influence of syntax and rhythm in cases where there are no strong sublexical cues to stress.

### Method

#### Participants

Twenty newly recruited undergraduate students from Royal Holloway, University of London, were paid £5 to participate in the study. Participants were monolingual native speakers of Southern British English and reported no visual, reading, or language difficulties.

#### Stimuli and design

Stimuli comprised a set of 80 disyllabic nonwords (five to seven-letters long) and a set of 80 sentence frames. Nonwords varied on the inclusion of sublexical stress cues that were associated with second-syllable stress: half of the nonwords contained such cues (cued), whereas the other half did not (non-cued). Cued nonwords consisted of the same 20 prefixed items with a long second vowel that were used in Experiments 1 and 3 (e.g., *preleal*, *resoud*, *preneem*) and a set of 20 new items. Non-cued nonwords consisted of the same 20 non-prefixed items with a short second vowel used in Experiments 1 and 2 (e.g., pralel, rosud, pranem) and a set of 20 new items. The new set of nonwords were constructed following the same constraints as in the previous experiments and were pairwise matched between conditions. Nonwords were group-wise matched on Coltheart’s N, *F*(1, 78) = 3.27, *p* = .08, (cued: *M* = 0.03, *SD* = 0.16; non-cued: *M* = 0.18, *SD* = 0.50) and the number of orthographic neighbors that take second-syllable stress, *F*(1, 78) = 1.00, *p* = .32. The nonword stimuli used in Experiment 4 are listed in Appendix D.

Nonwords were embedded in a set of sentences selected from Experiment 2 of Kelly and Bock (1988). These sentences contained six syllables with the embedded nonword occurring as the third and fourth syllable. The sentence stimuli varied on the syntactic context in which the embedded nonword would appear, so that a nonword appeared in a noun context in 40 sentences (e.g., “The blue *preleal* condensed”) and in a verb context in the remaining 40 sentences (e.g., “The rains *preleal* despair”). The rhythmic context in which the nonwords appeared was also varied, so that a nonword appeared in an iambic rhythmic context in half of the noun-context and verb-context sentences (e.g., “The blue *preleal* condensed” and “The rains *preleal* despair”) and in a trochaic rhythmic context in the other half of the noun-context and verb-context sentences (e.g., “Sell the *preleal* cheaply” and “Dogs will *preleal* kennels”). The sentence stimuli used in Experiment 4 are listed in Appendix E.

Four lists of experimental trials were created with different pseudorandomizations using the constraints that each nonword appeared (1) once in each list and (2) in all the grammatical and rhythmic context combinations across all lists. The presentation order within the lists was random, with the constraints that (a) pairwise-matched nonwords (e.g., *preleal*, *pralel*) appeared with a minimum distance of 20 experimental trials between each other, (b) no more than three sentences in which the nonword belonged to the same grammatical or rhythmic context appeared consecutively, and (c) sentences from the same conditions appeared in the same serial position in every list. List assignment was counterbalanced across participants. Following a practice session of four trials, each participant received all 80 sentences.

### Apparatus and procedure

The apparatus was the same as in the three previous experiments. However, the procedure in Experiment 4 slightly differed due to the nature of the task. On each trial, a fixation cross presented in the center of the screen for 500 ms was replaced with a sentence, which remained on the screen for 5,000 ms. Participants were asked to read aloud each sentence naturally and without hesitation, as if all of the items in the sentence were real words. The next trial followed after a 500-ms blank interval.

### Results

Disyllabic responses were classified as having received stress on the first or the second syllable, while hesitations and responses with anything other than two syllables were marked as erroneous and discarded (2.1% of all responses).^4^ The proportions of second-syllable stress in the different nonword conditions are presented in Table 6.[Table-anchor tbl6]

As for Experiments 1, 2, and 3, stress assignment data were analyzed using a generalized linear mixed-effects model. Our model included stressed syllable (first vs. second) as the dependent variable, sublexical cues (cues vs. no cues), syntactic context (nouns vs. verbs), rhythmic context (iambic vs. trochaic) and the triple interaction as fixed effects, and participants and items as crossed random effects. Bigram frequency contributed significantly to the model’s goodness of fit, χ^2^(1) = 3.78, *p* = .052, and was thus included in the final model as a covariate.

Results revealed a main effect of sublexical cues, χ^2^(1) = 98.44, *p* < .001), with nonwords containing sublexical cues receiving more second-syllable stress than those with no sublexical cues. As predicted by the fitted logistic regression model, the probability of second-syllable stress for items that contained no sublexical cues was 49%, whereas the corresponding probability for items with sublexical cues was 97%. There was also a main effect of syntactic context, χ^2^(1) = 82.77, *p* < .001, with nonwords placed in a verb context receiving more second-syllable stress than those placed in a noun context. The predicted probability of second-syllable stress for items in a noun position was 73%, whereas the corresponding probability for items in a verb position was 92%. Further, we found a main effect of rhythmic context, χ^2^(1) = 119.59, *p* < .001, with nonwords placed in an iambic context receiving more second-syllable stress than those placed in a trochaic context. The predicted probability of second-syllable stress for items in a trochaic context was 66%, whereas the predicted probability for items in an iambic context was 95%. The triple interaction among sublexical cues, syntactic context, and rhythmic context was not significant, χ^2^(1) = .15, *p* = .700. However, there was a significant interaction between syntactic and rhythmic context, χ^2^(1) = 12.65, *p* < .001. Post hoc comparisons (Tukey-adjusted) using the lsmeans (Lenth, 2016) package revealed a syntactic effect for nonwords that appeared in a trochaic context only (*z* = −9.00, *p* < .001) and a greater rhythm effect for nonwords that appeared in a noun context (*z* = −9. 47, *p* < .001) than for those that appeared in a verb context (*z* = −4.27, *p* = .006).

### Discussion

In line with previous findings (Kelly & Bock, 1988), the results from Experiment 4 revealed that both syntactic and rhythmic cues arising at the sentence-level influence the assignment of stress during reading aloud. Readers assigned more second-syllable stress to nonwords that were placed either in a verb or an iambic context compared with nonwords placed in a noun or a trochaic context, respectively. Furthermore, results showed a greater effect of rhythmic context for nonwords placed in a noun compared to a verb context, while syntax affected only nonwords placed in a trochaic context. These findings are most likely driven by the strong association between nouns and trochaic rhythm with first-syllable stress, which, as a result, exaggerated second-syllable stress differences. Nonwords placed in the noun trochaic context received the least proportion of second-syllable stress compared with all other contexts. This finding is consistent with the fact that nouns, which are more prominent in the speech stream (Sorensen, Cooper, & Paccia, 1978), are more likely to occupy strong positions in metrical patterns (i.e., trochaic context; Kelly & Bock, 1988). The strong stress bias for nouns is also reflected in the English language whereby approximately 90% of disyllabic nouns take first-syllable stress compared to the 67% of disyllabic verbs that take second-syllable stress (Howard & Smith, 2002).

More importantly, however, the results from Experiment 4 revealed the impact of sublexical cues on the assignment of stress during sentence reading. Irrespective of the syntactic and rhythmic context in which a nonword was placed, readers assigned more second-syllable stress to nonwords that contained sublexical cues (e.g., *preleal*) compared with nonwords that contained no sublexical cues (e.g., *pralel*). These findings are in line with those reported by Baker and Smith (1976) and Smith and Baker (1976), but they enhance our understanding of the relationship between word-level and sentence-level cues to stress assignment in a number of important ways. First, they reveal that sublexical cues to stress, which we have shown to impact stress placement in single-word reading, continue to operate in sentence reading. Second, they show that the influence of these lower-level stress cues functions independently of syntactic and rhythmic effects in sentence contexts. The relationship between lower-level cues operating at the word level and higher-level cues operating at the sentence level is additive. Finally, they reveal that even the combined effect of three key sublexical cues to stress does not extinguish the higher-level factors of syntax and rhythm, thus demonstrating the robustness of these contextual cues to stress in sentence reading.

The current findings appear to be inconsistent with those reported by Spinelli et al. (2016), who showed that the sublexical cue of stress neighborhood consistency to stress assignment in Italian is overridden by the presence of morpho-syntactic information. The present data do not allow us to understand the reason for this inconsistency. It may be that word-level distributional (word-ending) cues, which are influential in Italian, are particularly susceptible to higher-level contextual information. However, our experiments produced scant support for the role of word endings in English stress assignment. It is also worth noting that the nature of the higher-level information, and the method for introducing this, varied substantially between our study and that of Spinelli et al. (2016). Understanding the nature of cross-linguistic effects will be a matter for future research.

#### Model simulations

None of the computational accounts of reading aloud has anything to say about how sentence-level information can influence reading aloud. Thus, it is completely outside the scope of these models to simulate the effects of grammatical category and rhythm that we observed. This is an important limitation that should be addressed in the next generation of models. However, the fact that we observed an additive relationship between higher-level cues arising in sentence reading and lower-level cues arising in single-word reading means that we can assess how well these models capture the influences of the lower-level cues. Thus, we used the nonword stimuli presented in Experiment 4 to assess whether the performance of the RC00 algorithm (Rastle & Coltheart, 2000), the CDP++ model (Perry et al., 2010), and the SMA09 network (Ševa et al., 2009) on nonwords with and without sublexical cues approximates human reading performance, when nonwords are placed in particular syntactic (noun vs. verb) and/or rhythmic (trochaic vs. iambic) contexts. Table 7 displays the mean proportion of second-syllable stress to nonwords used in Experiment 4 for the three models under consideration.[Table-anchor tbl7]

The simulation data presented in Table 7 are consistent with the stimulations obtained from Experiments 1 through 3. All three models showed sensitivity to the presence of sublexical cues by assigning more second-syllable stress to nonwords that contained sublexical cues compared to nonwords without any sublexical cues. In line with previous simulations, the presence of prefixes in all nonwords that contained sublexical cues led the RC00 to grossly overestimate the assignment of second-syllable stress on these nonwords. In contrast, the sensitivity to sublexical cues exhibited by the CDP++ model and the SMA09 network was more moderated, and at least for SMA09, resembled the performance of human readers in the noun/trochaic context (see Table 6).

## General Discussion

It is virtually undisputed that the computation of sound-based codes is central to reading and reading acquisition (see, e.g., Frost, 1998; Perfetti, 2003; Rastle & Brysbaert, 2006; Rayner et al., 2001). Yet, theoretical and empirical research on how we translate orthography to phonology has been almost wholly restricted to monosyllables, thus ignoring the special challenges that emerge when polysyllables are considered, such as the computation of stress. This gap is especially notable in languages with a stress-free system, such as English, where stress has neither a fixed position within the word nor is marked by the use of diacritics. Fortunately, this narrow focus has begun to change with a growing body of empirical work (e.g., Arciuli & Cupples, 2006, 2007; Arciuli, Monaghan, & Ševa, 2010; Chateau & Jared, 2003; Jared & Seidenberg, 1990; Ktori et al., 2016; Mousikou et al., 2017; Sulpizio et al., 2015; Yap & Balota, 2009) and computational modeling (e.g., Jouravlev & Lupker, 2015a; Perry et al., 2010; Rastle & Coltheart, 2000; Ševa et al., 2009) focused on stimuli with more than one syllable.

The present study investigated the nature of sublexical knowledge that readers use to determine stress assignment during reading aloud disyllables in English. This knowledge is especially critical for the reading of unfamiliar words that are not stored in lexical memory and thus require the sublexical computation of stress. In particular, we examined whether three key sources of sublexical information, namely prefixation, the orthographic weight of a syllable, and vowel length, serve as cues to the assignment of stress. In a series of three experiments of single nonword reading, English adult readers read aloud carefully-constructed disyllabic nonwords in which we systematically varied the presence of these sublexical cues, and examined their effect on second-syllable stress. Our results revealed independent effects on the assignment of stress for each one of these cues: prefixed nonwords (e.g., *prelel*) received more second-syllable stress than non-prefixed nonwords (e.g., *pralel*); nonwords with a long vowel in their second syllable (e.g., *praleal*) received more second-syllable stress than nonwords with a short vowel in their second syllable (e.g., *pralell*); and nonwords with greater orthographic weight in their second syllable (e.g., *pralell*) received more second-syllable stress than nonwords with lesser orthographic weight in their second syllable (e.g., *pralel*). Previous literature has suggested the potential effects of these sublexical cues but has confounded the examination of some or all of these cues (e.g., Baker & Smith, 1976; Kelly et al., 1998; Rastle & Coltheart, 2000). Importantly, these effects were robust and consistent even after controlling for potential variation in similarity to existing words (OLD20), bigram frequency, and distributional characteristics of word endings (Arciuli & Cupples, 2006). The rigorous experimental approach employed in the present study enabled us to provide the first clear evidence of the individual impacts of key cues to stress assignment during English reading aloud.

In a fourth experiment, we examined how the effects of sublexical cues observed in single nonword reading interact with the higher-level contextual factors of syntax and rhythm that arise in sentence reading (Kelly & Bock, 1988). Stress assignment for sublexically cued nonwords (e.g., *preleal*) with a strong stress bias toward the second syllable was compared with that for non-cued nonwords (e.g., *pralel*) in sentences that varied the syntactic (i.e., nouns and verbs) and rhythmic (i.e., trochaic and iambic) contexts in which the nonwords appeared. Results replicated the influence of grammatical category and rhythm on nonword stress assignment first observed by Kelly and Bock (1988). More importantly, they revealed that the impact of sublexical cues on stress assignment persists in sentence contexts, and that these effects are additive with the higher-level effects of syntax and rhythm. Thus, even though the sentence frames provide very strong contextual cues to the stress assignment of the individual nonwords, this higher-level knowledge does not override the influence of lower-level sublexical cues. Conversely, even though the sublexically cued nonwords were heavily biased toward second syllable stress, this did not appear to influence the impact of the higher-level contextual cues. This work provides one of very few investigations of the relationship between sentence-level and word-level factors on reading aloud behavior.

### Rule-Based and Statistical-Learning Approaches to Stress Assignment

The present study demonstrates that the investigation of stress assignment provides a rich new source of evidence for adjudicating between opposing theoretical accounts of reading aloud that sometimes cannot be distinguished on the basis of empirical work in the monosyllabic domain. The assessment of the models’ general performance in capturing the modal stress assigned to the complete set of stimuli used in Experiments 1 through 3 by human participants revealed that the CDP++ model assigned stress correctly to 71% of cases (averaged across first-syllable and second-syllable stress) and performed slightly better than the RC00 algorithm, which predicted the human modal response in 69% of cases. The SMA09 network performed the worst by predicting human modal stress assignment in only 59% of cases. Deeper analyses of the factors underpinning model behavior revealed that none of the models perfectly captured the sensitivities that human readers show to the sublexical cues of prefixation, vowel length, and the orthographic weight of a syllable. The rule-based RC00 algorithm showed sensitivity to the sublexical cue of prefixation, but failed to show any sensitivity to the cues of vowel length and orthographic weight. Further, the RC00 algorithm grossly overestimated the effect of prefixation. The CDP++ model showed clear sensitivity to vowel length only, with limited sensitivity to prefixation and no evidence for sensitivity to orthographic weight. The SMA09 network came closest to simulating the human results, showing clear sensitivity to prefixation and subtle effects of vowel length and orthographic weight.

The very poor performance of the rule-based RC00 algorithm raises questions over whether stress assignment in reading aloud reflects rule-based behavior. Clearly, although participants in Experiments 1 through 3 showed robust effects of prefixation on stress assignment, the simulation data from the RC00 algorithm displayed in Tables 1 through 3 bears no resemblance to the human data. Although sensitive to prefixation, human readers did not treat prefixes in an all-or-none manner, as the hard-wired RC00 algorithm does. However, it is important to remember that the model simulations could be thought of as the performance of a single individual, rather than the performance of the average sample of participants (e.g., Mousikou et al., 2017). That is, although the model overestimated the impact of prefixation relative to the average performance across all participants, it is possible that different participants operate on the basis of different rules, and that the model behaved like one or more single participants. We thus inspected the behavior of individual subjects across Experiments 1 through 3 to ascertain whether any participant showed the all-or-none rule-based behavior exhibited by RC00 (i.e., near 100% second-syllable stress in the prefix-present conditions and near 0% second-syllable stress in the prefix-absent conditions). We could find no such participant. Thus, although the RC00 algorithm captures human readers’ sensitivity to prefixes in stress assignment, it seems clear that it does not capture this information in the same manner as human readers do.

Both of the statistical learning models performed better than the rule-based model, although the performance of SMA09 was closest to the behavior of human readers. Because both of these models capitalize on statistical regularities in their training sets to learn the spelling-to-stress mapping, it is interesting to consider why the performance of these models differed. We believe that two important differences between the models can account for the divergent performance. The first is related to the way the orthographic input is coded in these models. The input to the CDP++ model contains a highly structured letter template that segments stimuli in each syllable into onset and rime units, whereas the SMA09 network aligns all orthographic inputs to the left and on the first letter. These alignment differences can impact the nature of the spelling-to-stress associations that are learned in these models. For example, the CDP++ model is able to learn strong systematic associations between the second vowel and the stress pattern of a disyllabic stimulus because the input template specifies the location of the second vowel. It is far more difficult for the SMA09 network to learn this association because the left-aligned nature of its input means that the second vowel will be only partially aligned across the training set. We believe that this difference in input coding may account for the fact that CDP++ showed clear sensitivity to vowel length while SMA09 showed only subtle sensitivity to this sublexical cue.

The second important difference between the CDP++ model and the SMA09 network relates to the training set to which the models were exposed. The CDP++ model was trained using both monosyllabic and disyllabic word databases, whereas the SMA09 network was trained only on a disyllabic word database. This difference has important implications for the models’ success in simulating the impact of prefixation on stress assignment. Despite the structured input template of the CDP++ model described above, both of these models align onsets to the left. This means that both models should be able to develop an association between groups of initial letters corresponding to prefixes and second syllable stress. However, only the SMA09 model showed sensitivity to this factor. We believe that this difference can be explained by these models’ different training sets. Specifically, because many monosyllabic words such as *red*, *press*, and *desk* begin with letter sequences that correspond to prefixes, the inclusion of such words in the CDP++ model training set may have prevented that model from forming a strong association between groups of letters corresponding to prefixes and second-syllable stress. Because the SMA09 model did not include these monosyllabic words in its training set, the formation of such an association for that model might have been more straightforward. Indeed, the SMA09 network’s sensitivity to prefixation was greater than the sensitivity shown by human readers. We anticipate that the clear effect of prefixation in the SMA09 network could disappear if the model were trained on a word corpus containing both monosyllables and disyllables.^5^

The relative success of the CDP++ and SMA09 models indicates that stress assignment in English reading aloud may reflect sensitivity to statistical information in the mapping between spelling and stress. Although it is clear that nonwords were often produced with second-syllable stress, and that this was predictable on the basis of the sublexical cues investigated, it is also clear that participants revealed a bias toward initial stress. Only when two or more sublexical cues were combined did the incidence of second-syllable stress go over 50%. Thus, while the sublexical assignment of stress is not as simple as producing the most frequent stress pattern, it seems that the general distribution of stress patterns in the language is influential. It could be that adult English readers begin with an initial-syllable stress bias, and that the sublexical cues that we have investigated have the effect of pulling stress away from the initial syllable. It is also possible that the sublexical cues that we have identified operate in a statistical manner. To assess this, we asked whether particular prefixes provide more powerful markers of second-syllable stress based on the consistency with which they are associated with second-syllable stress in the writing system. Our analyses revealed no relationship between the proportion of words with a particular prefix that take second-syllable stress and the mean proportion of second-syllable stress that readers assigned on nonwords with the same prefix (*r* = .141, *p* = .282, based on data collapsed across Experiments 1 through 3).^6^ However, it is important to acknowledge the limited range of prefixes studied in our experiments. Finally, our experiments yielded only very weak evidence for an influence of word endings on stress assignment (cf. Arciuli & Cupples, 2006). This continuous variable did not modulate any of the sublexical cues studied, and was reliable in only one of the four experiments. Overall, our results favor an account of stress assignment based on statistical learning as opposed to all-or-none rules, but further research will be necessary to determine precisely the nature of this statistical learning.

### Bayesian Approaches to Stress Assignment

Recently, Jouravlev and Lupker (2015a) have considered an alternative approach to stress assignment in reading aloud. Although this is not a processing model like those described earlier, we consider it in the present paper because it provides an alternative way of thinking about the summation of different sublexical cues in reading aloud behavior. Jouravlev and Lupker (2015a) put forward the idea that stress assignment can be viewed as a problem of Bayesian probabilistic inference in which readers evaluate the likelihood of potential stress patterns when making a stress assignment decision. This decision is accomplished by considering prior beliefs about the likelihood of stress patterns in a language and adjusting them in the light of evidence for a specific stress pattern derived from the letter string that is being read aloud. Evidence for stress could be lexical (i.e., a retrieved stress pattern from memory) or sublexical (i.e., sublexical cues), with the likelihood of using lexical versus sublexical evidence based on the ease of lexical access for a particular item. For example, it would be more likely to use lexical evidence for high frequency words, whereas it would be more likely to use sublexical evidence for unfamiliar words and nonwords.

The principles of this Bayesian account may appear similar to those of the connectionist-learning account since both of these approaches predict stress by drawing on the statistical regularities of the writing system. However, these two approaches differ in two key respects. First, the Bayesian account uses the distributional knowledge about stress pattern as a baseline to reflect a prior belief about the likelihood that a word has a specific stress pattern. In contrast, the connectionist-learning account assumes no such prior knowledge. The second, and perhaps more important difference, between the Bayesian and the connectionist-learning accounts relates to the number of sublexical sources of information considered in the computation of stress. Although the connectionist-learning approach does not propose any specific sublexical cues to stress assignment, the Bayesian account specifies a limited number of sublexical sources of evidence and assumes that these are sufficient to provide predictions of stress patterns. In this way, specific predictions about relevant sources of evidence are virtually hard-coded into the Bayesian account, which is not the case in the connectionist-learning approach.

Using this approach, Jouravlev and Lupker (2015a) demonstrated that the posterior probabilities of stress patterns computed for Russian disyllabic words and nonwords correlated highly with the stress patterns that Russian readers assigned to these items. Here, we evaluated whether the process of stress assignment in English can be also explained within this Bayesian framework. Specifically, we computed the probabilities of stress patterns for the nonword stimuli used in Experiments 1, 2, and 3, and then we compared them to the human stress data. For each nonword, we estimated the posterior probability of second-syllable stress by considering the presence of a prefix, a long vowel in the second syllable, and the orthographic weight of the second syllable (i.e., expressed as the difference in letters between the second and the first syllable).

The computations of the prior probabilities of stress patterns in the English language as well as the likelihood of evidence based on the three sublexical cues under investigation were derived from a corpus of 42,980 English disyllabic words in the CELEX database (Baayen et al., 1995), following the removal of abbreviations, unique entities, and items whose orthographic syllabification did not contain two syllables. In the absence of any sublexical cue in a nonword, the posterior probability of second-syllable stress was equal with the prior probability derived from the distribution of second-syllable stress in the corpus. For example, the likelihood of second-syllable stress for the nonword *mesdut* is .27 because 27% of the words in the corpus are stressed on the second-syllable. In the presence of a sublexical cue, the posterior probability of second-syllable stress was computed by adjusting the prior probability to take into account the proportion of all the words in the corpus that contain the given sublexical cue and take second-syllable stress (see Jouravlev & Lupker, 2015a, p.1176, for the exact formulae). For example, the likelihood of second-syllable stress for the nonword *misdut* is .58, because the presence of the prefix *mis-* increased the prior likelihood of this nonword receiving second-syllable stress compared to when such a prefix was absent (i.e., *mesdut*). Finally, in the presence of multiple sublexical cues, the posterior probability of second-syllable stress was computed in separate stages. At every stage, the posterior probability of second-syllable stress was calculated by using the prior probability of second-syllable stress, as determined by the presence of a previously considered sublexical cue, and by adjusting it in the light of the evidence provided by a newly considered sublexical cue. For example, the posterior probability of the nonword *misdoot* is .72 due to the presence of *mis-*, the long vowel /u/ in the second-syllable, and the higher orthographic weight of the second syllable compared to the first syllable.^7^

The estimated probabilities of second-syllable stress for our nonword stimuli correlated significantly with the proportion of second-syllable stress that readers assigned to these nonwords (Experiment 1: *r*[76] = .51, *p* < .001; Experiment 2: *r*[80] = .35, *p* < .001; Experiment 3: *r*[80] = .59, *p* < .001; across all experimental stimuli: *r*[120] = .49, *p* < .001). We used the Bayesian probabilities derived from the equations provided by Jouravlev and Lupker (2015a) to compute a binary variable expressing whether the model predicted first- or second-syllable stress. We then compared this binary variable to the modal stress assigned by human readers on the complete set of stimuli. This analysis revealed that the Bayesian account predicted the modal human stress for 73% of the items that were assigned first-syllable stress, and for 43% of the items that were assigned second syllable stress (*d′* = 0.79; *c* = −.23; see Table 4). Thus, on the basis of just three sources of sublexical knowledge and the overall distribution of stress pattern in English disyllables, the Bayesian account approximated the stress performance revealed by the CDP++ model. However, there remains considerable work to do in order to express this theory in the form of a full processing model.

### Integrating Sentence-Level Cues in Models of Reading Aloud

Models of reading aloud describe only the representations and processes operating at the single-word level. They have nothing to say about how higher-level information is processed in sentence contexts, or how such information may constrain processes at the level of the single word. Because we rarely encounter words in isolation, it could be said that these models lack ecological validity. This argument has often been put forward to support the need to study natural reading in sentence contexts (e.g., Rayner & Reichle, 2010), although models in this domain have nothing to say about the computation of phonology. To a certain extent, our work provides some vindication of models of reading aloud that focus on the single word. For the first time, our work shows that the factors that underpin processing at the single-word level also operate when letter strings are read aloud in sentences. Moreover, these word-level factors do not interact with higher-level factors that arise due to the ongoing interpretation of a sentence. These data therefore provide confidence in the validity of research on single-word reading aloud. The observations that arise in single-word reading are not uninteresting manifestations of the single-word paradigm.

Nevertheless, our data also expose the limitations of computational models of reading aloud. Our data suggest that multiple sources of information are integrated while sentences are read aloud. Thus, the pronunciation of a single word in a sentence context is influenced not only by information about the word itself, but also by the syntactic and rhythmic frame. Ultimately, a complete model of reading aloud will have to describe the mechanisms that give rise to these additional contextual influences. Developing such a model is surely a challenge for future research. This work will require an understanding of (a) how sentence-level information might influence the computation of phonology and (b) how this higher-level information combines with word-level information to resolve the final pronunciation. With respect to (b), we note with interest the view that sentence-level information can be construed as influencing the prior probability within a Bayesian framework for understanding reading (Norris, 2006). If stress assignment were conceived within such a framework (e.g., Jouravlev & Lupker, 2015a), one could argue that ongoing syntactic and rhythmic analysis of the sentence contributes to the accumulation of evidence regarding the stress patterns of upcoming words.

### Implications for Developing Readers and Clinical Populations

Our findings have important scientific and pedagogical implications for developing readers. The last 15 years have seen major strides in policy and practice in the teaching of reading. There is broad consensus that reading instruction programs that emphasize the alphabetic principle through phonics are most effective in the first stages of learning to read (see Rayner et al., 2001; Rose, 2006, for review). The use of phonics in initial reading instruction is described in the Common Core, and has been instantiated as a statutory requirement in all state schools in England and Wales. Systematic phonics programs focus on the explicit teaching of the relationship between graphemes and phonemes. However, the phonic relationships that are taught pertain most strongly to monosyllables. None of these programs considers the special challenges arising when polysyllabic words are considered, such as syllabification, vowel reduction, and stress (e.g., which types of cues are associated with different stress patterns). However, such knowledge is necessary as children begin to encounter longer, multisyllabic words in reading acquisition (Arciuli, Monaghan, & Ševa, 2010). Moreover, children’s prosodic ability seems to be associated with reading fluency and comprehension skill (Holliman, Mundy, Wade-Woolley, Wood & Bird, 2017; Miller & Schwanenflugel, 2008; Whalley & Hansen, 2006). Thus, it would be of considerable benefit to children learning to read to have explicit instruction on stress assignment as part of their phonics programs. It is clear that part of the challenge has been that the constraints that govern the relationship between spellings and stress have not been previously articulated. We therefore anticipate that the empirical work reported in this article will provide the first step toward developing these reading instruction programs more fully.

Our findings may also be relevant to the clinical assessment of reading impairments. Participants with developmental and acquired reading difficulties are typically assessed using nonword reading aloud tasks (e.g., Castles & Coltheart, 1993; Rack, Snowling, & Olson, 1992). Intriguingly, these tests are not limited to monosyllables, raising the question of how responses to polysyllabic nonwords are scored (in particular, the decision of where to place the stress). In the absence of a detailed understanding of the sublexical cues to stress assignment, we suspect that pronunciations are deemed correct as long as they have adequate segmental information, with information about stress assignment being ignored. This state of affairs is clearly nonoptimal as our lack of knowledge limits the depth with which these populations can be assessed. Further, there is evidence that both developmental (e.g., Dulay & Hanley, 2015; Paizi, Zoccolotti, & Burani, 2011) and acquired (e.g., Ktori et al., 2016) dyslexics make stress errors when reading words aloud; it seems likely that sublexical cues to stress assignment will influence such individuals’ nonword reading aloud performance. Our findings provide the first step toward a full description of the sublexical computation of stress in reading aloud, thus paving the way for the fuller assessment of these individuals in future.

## Conclusion

The present study has begun to delineate the individual impacts of particular sublexical cues on stress assignment in reading aloud. Our work establishes that readers use prefixation, vowel length, and the orthographic weight of a syllable as independent sources of sublexical information in stress assignment. Our simulations reveal that the rule-based model of stress assignment (Rastle & Coltheart, 2000) provides an inadequate account of human performance, and further inspection of the performance of individual participants showed no instances of rule-based behavior (at least on the rules proposed by RC00). Simulations using distributed-connectionist (Perry et al., 2010; Ševa et al., 2009) and Bayesian approaches (Jouravlev & Lupker, 2015a) were more promising, and indicate that stress assignment in reading aloud may be best conceived within a statistical learning or probabilistic framework. Our work has also begun to bridge research on single-word reading aloud with constraints arising at the sentence level. Critically, our empirical work revealed that sublexical cues to stress assignment are additive with sentence-level rhythmic and syntactic cues to stress assignment. This result provides reassurance of the validity of models that describe reading at the level of isolated words. However, the fact that sentence-level rhythmic and syntactic information impacts on stress assignment of individual words also exposes important weaknesses in our theoretical conceptualisations of reading aloud. Even though substantial further work will be required to develop a fully implemented model of reading aloud that moves beyond monosyllables in isolated contexts, the research presented in this article provides important initial constraints on the characteristics of that final model. We have argued that this ongoing theoretical work is critical for the development of effective assessment tools for the characterization of acquired and developmental reading impairment, as well as the development of reading instruction programs that are relevant for the vast majority of words in our vocabulary.

## Figures and Tables

**Table 1 tbl1:** Human and Model Mean Proportions of Second-Syllable Stress as a Function of Prefixation and Second-Vowel Length in Experiment 1 (Mean Standard Error for the Human Data in Parentheses)

Condition	Human data	RC00	CDP++	SMA09
Non-prefixed short vowel (e.g., *pralel*)	.30 (.06)	0	0	.10
Non-prefixed long vowel (e.g., *praleal*)	.40 (.07)	.05	.55	.25
Prefixed short vowel (e.g., *prelel*)	.40 (.07)	.95	.10	.55
Prefixed long vowel (e.g., *preleal*)	.56 (.07)	1.00	.60	.75
*Note.* CDP++ = connectionist dual process model (Perry, Ziegler, & Zorzi, 2010); RC00 = rule-based algorithm (Rastle & Coltheart, 2000); SMA09 = print-to-stress model (Ševa, Monaghan, & Arciuli, 2009).				

**Table 2 tbl2:** Human and Model Mean Proportions of Second-Syllable Stress as a Function of Prefixation and the Orthographic Weight of the Second Syllable in Experiment 2 (Mean Standard Error for the Human Data in Parentheses)

Condition	Human data	RC00	CDP++	SMA09
Non-prefixed light syllable (e.g., *pralel*)	.17 (.05)	0	0	.10
Non-prefixed heavy syllable (e.g., *pralell*)	.25 (.06)	0	0	.15
Prefixed light syllable (e.g., *prelel*)	.28 (.06)	.95	.10	.55
Prefixed heavy syllable (e.g., *prelell*)	.36 (.07)	.95	.10	.60
*Note.* CDP++ = connectionist dual process model (Perry, Ziegler, & Zorzi, 2010); RC00 = rule-based algorithm (Rastle & Coltheart, 2000); SMA09 = print-to-stress model (Ševa, Monaghan, & Arciuli, 2009).				

**Table 3 tbl3:** Human and Model Mean Proportions of Second-Syllable Stress as a Function of Prefixation and Second-Vowel Length, With the Orthographic Weight of the Second Syllable Controlled, in Experiment 3 (Mean Standard Error for the Human Data in Parentheses)

Condition	Human data	RC00	CDP++	SMA09
Non-prefixed short vowel (e.g., *pralell*)	.32 (.08)	0	0	.15
Non-prefixed long vowel (e.g., *praleal*)	.39 (.08)	.05	.55	.25
Prefixed short vowel (e.g., *prelell*)	.46 (.08)	.95	.10	.60
Prefixed long vowel (e.g., *preleal*)	.52 (.08)	1.00	.60	.75
*Note.* CDP++ = connectionist dual process model (Perry, Ziegler, & Zorzi, 2010); RC00 = rule-based algorithm (Rastle & Coltheart, 2000); SMA09 = print-to-stress model (Ševa, Monaghan, & Arciuli, 2009).				

**Table 4 tbl4:** Contingency Tables Showing the Distribution of Stress Pattern Assigned by Human Readers and Predicted by the RC00 Algorithm, the CDP++ Model, the SMA09 Network, and the Bayesian Account to the Items Presented in Experiments 1, 2, and 3

	RC00		CDP++		SMA09		Bayesian account		
Human stress	First syllable	Second syllable	First syllable	Second syllable	First syllable	Second syllable	First syllable	Second syllable	Total
First syllable	54	36	79	11	58	32	66	24	90
Second syllable	7	23	14	16	14	16	17	13	30
Total	61	59	93	27	72	48	83	37	120
*Note.* Values presented in raw numbers of items. CDP++ = connectionist dual process model (Perry, Ziegler, & Zorzi, 2010); RC00 = rule-based algorithm (Rastle & Coltheart, 2000); SMA09 = print-to-stress model (Ševa, Monaghan, & Arciuli, 2009); Bayesian account = probabilistic inference account (Jouravlev & Lupker, 2015a).									

**Table 5:** Logistic Regression Analyses on Second-Syllable Stress Data for the RC00 Algorithm, the CDP++ Model, and the SMA09 Network in Experiments 1, 2, and 3 (Unstandardized Regression Coefficients (B) With Their Standard Errors (SE) in Parentheses)

	RC00	CDP++	SMA09
Predictor variable	*B* (*SE*)	*B* (*SE*)	*B* (*SE*)
Experiment 1: Prefixation and second-vowel length^a^			
Prefixation	7.33 (1.43)***	.52 (.59)^*ns*^	2.40 (.87)**
Second-vowel length	—	3.28 (.80)***	1.10 (.91)^*ns*^
Prefixation × Second-Vowel length	—	—	−.20 (1.14)^*ns*^
Experiment 2: Prefixation and orthographic weight^b^			
Prefixation	Complete separation	—	2.40 (.87)**
Orthographic weight	—	—	.46 (.97)^*ns*^
Prefixation × Orthographic Weight	—	—	−.25 (1.17)^*ns*^
Experiment 3: Prefixation and second-vowel length (orthographic weight controlled)^c^			
Prefixation	7.33 (1.43)***	.52 (.59)^*ns*^	2.14 (.77)**
Second-vowel length	—	3.28 (.80)***	.64 (.81)^*ns*^
Prefixation × Second-Vowel Length	—	—	.06 (1.06)^*ns*^
*Note*. Dashes indicate that the variable was not included in the final regression model. CDP++ = connectionist dual process model (Perry, Ziegler, & Zorzi, 2010). RC00 = rule-based algorithm (Rastle & Coltheart, 2000). SMA09 = print-to-stress model (Ševa, Monaghan, & Arciuli, 2009).			
^a^ Experiment 1. RC00: *R*^2^ = .8 (Hosmer & Lemeshow), Model χ^2^(1) = 92.20, *p* < .001; CDP++: *R*^2^ = .3 (Hosmer & Lemeshow), Model χ^2^(2) = 29.72, *p* < .001; SMA09: *R*^2^ = .2 (Hosmer & Lemeshow), Model χ^2^(3) = 22.93, *p* < .001. ^b^ Experiment 2 SMA09: *R*^2^ = .2 (Hosmer & Lemeshow), Model χ^2^(3) = 19.23, *p* < .001. ^c^ Experiment 3. RC00:*R*^2^ = .8 (Hosmer & Lemeshow), Model χ^2^(1) = 92.20, *p* < .001; CDP++: *R*^2^ = .3 (Hosmer & Lemeshow), Model χ^2^(2) = 29.72, *p* < .001; SMA09 *R*^2^ = .2 (Hosmer & Lemeshow), Model χ^2^(3) = 20.83, *p* < .001.			
** *p* < .01. *** *p* < .001. n.s. = non-significant.			

**Table 6 tbl6:** Mean Proportions of Second-Syllable Stress on Nonwords With and Without Sublexical Cues to Stress Assignment as a Function of Syntactic and Rhythmic Context in Experiment 4 (Mean Standard Error in Parentheses)

Syntactic context	Rhythmic context	Non-cued nonwords	Cued nonwords
Noun	Trochaic	.18 (.05)	.69 (.06)
	Iambic	.55 (.07)	.97 (.01)
Verb	Trochaic	.49 (.06)	.90 (.03)
	Iambic	.71 (.06)	.97 (.01)

**Table 7 tbl7:** Model Mean Proportions of Second-Syllable Stress on Nonwords With and Without Sublexical Cues in Experiment 4

Condition	RC00	CDP++	SMA09
Non-cued nonwords (e.g., *pralel*)	0	.03	.13
Cued nonwords (e.g., *preleal*)	1	.70	.80
*Note.* CDP++ = connectionist dual process model (Perry, Ziegler, & Zorzi, 2010); RC00 = rule-based algorithm (Rastle & Coltheart, 2000); SMA09 = print-to-stress model (Ševa, Monaghan, & Arciuli, 2009).			

## Footnotes

1 We chose not to consider the impact of word beginnings because this work has been developed much less fully than that on word endings, and the existing evidence pertaining to word beginnings is very weak. Though Arciuli and Cupples (2007) provided a preliminary sketch of this concept in a book chapter, their empirical work fully confounded word beginnings with prefixation. It is also noteworthy that Jouravlev and Lupker (2015b) found no evidence for the impact of word beginnings on stress assignment in Russian.

2 Two independent raters with no previous training or experience in stress judgments and blind to the conditions and purposes of the present experiments were also asked to judge the assignment of stress for a subset of participants in each of the four experiments. The interrater reliability between K.R. and each of the independent raters ranged from substantial (κ = .60–.80) to almost perfect (κ > .80). The interrater reliability between K.R. and the first rater across the four experiments was κ = .80, *p* < .001 in Experiment 1, κ = .87, *p* < .001 in Experiment 2, κ = .72, *p* < .001 in Experiment 3, and κ = .79, *p* < .001 in Experiment 4. The interrater reliability between K.R. and the second rater was κ = .91, *p* < .001 in Experiment 1, κ = .93, *p* < .001 in Experiment 2, κ = .75, *p* < .001 in Experiment 3, and κ = .71, *p* < .001 in Experiment 4.

3 The analysis of the simulation data that we report did not include any covariates. This was because due to the small sample size, the complexity of the statistical model compromised the reliability of the results. However, even when such covariates (i.e., OLD20, mean bigram frequency, ending frequency, ending-to-second-syllable-stress proportion) were included in the statistical models, the pattern of results remained identical.

4 Judgment of stress placement was performed both with the nonwords embedded in the sentence (in-sentence scoring) and with the nonwords in isolation after they had been extracted from their sentence context (in-isolation scoring). The reliability across different methods of scoring was substantial (κ = .78, *p* < .001) and data analyses revealed the same pattern of results. We chose to report the data from the in-sentence scoring because the pronunciation of the extracted nonwords was occasionally distorted by influences from surrounding sounds, deeming the in-isolation scoring less reliable.

5 We are grateful to Padraic Monaghan for this insight regarding the performance of the SMA09 network.

6 A separate analysis was performed to assess the impact of distributional information of the non-prefix beginnings used in the experimental stimuli on second-syllable stress assignment. Results revealed a nonsignificant correlation between the proportion of words with a particular nonprefix beginning that take second-syllable stress and the mean proportion of second-syllable stress that readers assigned on nonwords with the same non-prefix (*r* = .057, *p* = .666; across all participants from Experiments 1 through 3). This result provides further evidence that skilled readers of English show no sensitivity to the distributional information of word beginnings during stress assignment.

7 In those cases where multiple sublexical cues were present in a nonword, the evidence from each cue was entered into the equation sequentially, beginning with the most informative cue in the CELEX database (i.e., prefixation) and ending with the least informative cue (i.e., orthographic weight). In the cases of overlapping evidence (i.e., where both vowel length and orthographic weight were present in a nonword), the evidence provided by the orthographic weight of the syllable was corrected, so as to exclude the evidence already accounted for by the long vowel of the second syllable in the computation of posterior probabilities (as per Jouravlev & Lupker, 2015a).

## A Nonword Stimuli Used in Experiment 1

**Table:**

Non-prefixed short vowel	Non-prefixed long vowel	Prefixed short vowel	Prefixed long vowel
dopom	dopoam	depom	depoam
dorof	doroaf	derof	deroaf
dorus	dorous	derus	derous
dotep	doteap	detep	deteap
dozat*	dozait*	dezat*	dezait*
mesdut	mesdoot	misdut	misdoot
mesfod	mesfoad	misfod	misfoad
pradus	pradous	predus	predous
praket	prakeet	preket	prekeet
pralel	praleal	prelel	preleal
prapem	prapeam	prepem	prepeam
prasof	prasoaf	presof	presoaf
pratob	pratoab	pretob	pretoab
pravun	pravoun	prevun	prevoun
prazan	prazain	prezan	prezain
rojat	rojait	rejat	rejait
ronen	roneen	renen	reneen
rosom	rosoam	resom	resoam
rosud	rosoud	resud	resoud
rovon	rovoan	revon	revoan
*Note.* * = Item not presented.			

## B Nonword Stimuli Used in Experiment 2

**Table:**

Non-prefixed light syllable	Non-prefixed heavy syllable	Prefixed light syllable	Prefixed heavy syllable
dopom	dophom	depom	dephom
dorof	doroff	derof	deroff
dorus	doruss	derus	deruss
dotep	dothep	detep	dethep
dozat	dozath	dezat	dezath
mesdut	mesduth	misdut	misduth
mesfod	mesfodd	misfod	misfodd
pradus	praduss	predus	preduss
praket	praketh	preket	preketh
pralel	pralell	prelel	prelell
prapem	praphem	prepem	prephem
prasof	prasoff	presof	presoff
pratob	prathob	pretob	prethob
pravun	pravunn	prevun	prevunn
prazan	prazang	prezan	prezang
rojat	rojath	rejat	rejath
ronen	roneng	renen	reneng
rosom	roshom	resom	reshom
rosud	roshud	resud	reshud
rovon	rovong	revon	revong

## C Nonword Stimuli Used in Experiment 3

**Table:**

Non-prefixed short vowel	Non-prefixed long vowel	Prefixed short vowel	Prefixed long vowel
dophom	dopoam	dephom	depoam
doroff	doroaf	deroff	deroaf
doruss	dorous	deruss	derous
dothep	doteap	dethep	deteap
dozath	dozait	dezath	dezait
mesduth	mesdoot	misduth	misdoot
mesfodd	mesfoad	misfodd	misfoad
praduss	pradous	preduss	predous
praketh	prakeet	preketh	prekeet
pralell	praleal	prelell	preleal
praphem	prapeam	prephem	prepeam
prasoff	prasoaf	presoff	presoaf
prathob	pratoab	prethob	pretoab
pravunn	pravoun	prevunn	prevoun
prazang	prazain	prezang	prezain
rojath	rojait	rejath	rejait
roneng	roneen	reneng	reneen
roshom	rosoam	reshom	resoam
roshud	rosoud	reshod	resoud
rovong	rovoan	revong	revoan

## D Nonword Stimuli Used in Experiment 4

**Table:**

Non-cued	Cued	Non-cued	Cued
dopom	depoam	dokef	dekeaf
dorof	deroaf	dokem	dekeem
dorus	derous	dopeb	depeeb
dotep	deteap	dopof	depoaf
dozat	dezait	mesbok	misboak
mesdut	misdoot	mesfep	misfeep
mesfod	misfoad	mesgef	misgeef
pradus	predous	mesgok	misgoak
praket	prekeet	pradez	predeez
pralel	preleal	pragom	pregoam
prapem	prepeam	pramef	premeef
prasof	presoaf	pranem	preneem
pratob	pretoab	prazod	prezoad
pravun	prevoun	rojop	rejoap
prazan	prezain	romep	remeap
rojat	rejait	ronuf	renoof
ronen	reneen	ropef	repeef
rosom	resoam	ropum	repoum
rosud	resoud	roseb	reseeb
rovon	revoan	rotud	retood

## E Sentence Stimuli Used in Experiment 4 Across the Different Experimental Conditions

**Table:**

	Syntactic context	
Rhythmic context	Noun	Verb
Iambic	The red _____ emerged.	The birds _____ concern.
Iambic	The pure _____ dissolved.	The jets _____ Japan.
Iambic	The old _____ expired.	The pills _____ disease.
Iambic	The sick _____ revived.	The costs _____ demand.
Iambic	The blue _____ condensed.	The rains _____ despair.
Iambic	The young _____ enrolled.	The drugs _____ raccoons.
Iambic	The lost _____ returned.	The songs _____ guitars.
Iambic	The small _____ believed.	The clerks _____ cigars.
Iambic	The long _____ commenced.	The queens _____ estates.
Iambic	The strong _____ attacked.	The drinks _____ Annette.
Iambic	The proud _____ refused.	The pins _____ balloons.
Iambic	The new _____ resumed.	The hats _____ Eileen.
Iambic	The calm _____ awoke.	The schools _____ careers.
Iambic	The strange _____ arrived.	The kids _____ cartoons.
Iambic	The short _____ convened.	The goats _____ plateaus.
Iambic	The fat _____ matured.	The beers _____ adults.
Iambic	The free _____ conformed.	The stores _____ receipts.
Iambic	The lone _____ remained.	The states _____ frontiers.
Iambic	The ill _____ regressed.	The roads _____ cement.
Iambic	The true _____ arrived.	The dukes _____ conceit.
Trochaic	Slice the _____ slowly.	Gold will _____ kingdoms.
Trochaic	Chase the _____ swiftly.	Steam will _____ engines.
Trochaic	Make the _____ shapely.	Salt will _____ oceans.
Trochaic	Watch the _____ calmly.	Bells will _____ towers.
Trochaic	Sell the _____ cheaply.	Dogs will _____ kennels.
Trochaic	Take the _____ flatly.	Loans will _____ bankers.
Trochaic	Cut the _____ thickly.	Meat will _____ muscles.
Trochaic	Drop the _____ quaintly.	Tea will _____ dresses.
Trochaic	Call the _____ shortly.	Stars will _____ heaven.
Trochaic	Kiss the _____ nicely.	Wine will _____ parties.
Trochaic	Use the _____ proudly.	Planes will _____ pilots.
Trochaic	Sing the _____ sweetly.	Boats will _____ sailors.
Trochaic	Solve the _____ strangely.	Goals will _____ mountains.
Trochaic	Play the _____ poorly.	Psalms will _____ prophets.
Trochaic	Press the _____ flatly.	Cheese will _____ shepherds.
Trochaic	Run the _____ strictly.	John will _____ countries.
Trochaic	Join the _____ freely.	Blood will _____ jackets.
Trochaic	Try the _____ justly.	Golf will _____ athletes.
Trochaic	Speak the _____ truly.	Dirt will _____ sneakers.
Trochaic	Read the _____ simply.	Hens will _____ roosters
